# Host transcriptomic analysis reveals a defective intracellular environment that limits SARS-CoV-2 replication in CFTR-deficient airway epithelium

**DOI:** 10.3389/fcimb.2026.1754083

**Published:** 2026-03-25

**Authors:** Anna Lagni, Virginia Lotti, Riccardo Cecchetto, Emil Tonon, Erica Diani, Asia Palmisano, Pier Paolo Piccaluga, Matteo Calgaro, Nicola Vitulo, Claudio Sorio, Davide Gibellini

**Affiliations:** 1 Microbiology Section, Department of Diagnostic and Public Health, University of Verona, Verona, Italy; 2 Unità Operativa Complessa (UOC) Micorbiology, Azienda Ospedaliera Universitaria Integrata (AOUI) Verona, Verona, Italy; 3 Biobank of Research, Istituto di Ricovero e Cura a Carattere Scientifico (IRCCS) Azienda Ospedaliera-Universitaria di Bologna Policlinico di S. Orsola, Bologna, Italy; 4 Department of Medical and Surgical Sciences, Bologna University School of Medicine, Bologna, Italy; 5 Department of Biotechnology, University of Verona, Verona, Italy; 6 General Pathology Section, Department of Medicine, University of Verona, Verona, Italy

**Keywords:** CFTR dysfunction, cystic fibrosis, host-virus interaction, SARS-CoV-2, transcriptome

## Abstract

Cystic fibrosis (CF) is characterized by chronic airway inflammation, yet clinical observations have revealed more favorable COVID-19 outcomes than originally predicted. Several studies demonstrated a significant decrease of SARS-CoV-2 replication in CF-mutated bronchial cells suggesting that CFTR dysfunction may interfere with viral replication, though the underlying mechanisms remain unclear. To elucidate these mechanisms we performed transcriptomic profiling of SARS-CoV-2-infected bronchial epithelial cells with wild-type (WT) or mutated CFTR, using both immortalized and primary airway models. RNA-seq was performed on WT and CF cellular models before and at 24, 48, and 72-hours post-infection. The differentially expressed genes (DEGs) were defined as genes with a log2 fold change>1 between groups (p<0.05) and significant DEGs were subjected to Gene Ontology and KEGG enrichment analysis (p<0.05). Our results reveal that CFTR deficiency impairs SARS-CoV-2 replication not by altering receptor availability (e.g., *ACE2, TMPRSS2*), but through widespread intracellular remodeling defects. CF cells failed to activate key antiviral and inflammatory responses, including interferon signaling, AP-1 transcriptional complex, and IL-6-mediated pathways. Furthermore, they exhibited defective unfolded protein response, altered calcium signaling, and disrupted ER-mitochondrial communication. Crucially, pH dysregulation and impaired expression of V-ATPase subunits and autophagy-related genes hindered vesicle acidification, double-membrane vesicle formation, and viral assembly. These intrinsic alterations also blunted virus-induced senescence programs. Collectively, our findings indicate that CF cellular environment is intrinsically unfavorable to SARS-CoV-2, limiting its replication and propagation. This study provides a mechanistic basis for the reduced viral burden observed in CF and highlights intracellular pH regulation and organelle homeostasis as potential therapeutic targets against SARS-CoV-2 infection.

## 1 Introduction

Cystic fibrosis (CF) is a multisystem genetic disorder primarily affecting the lungs, pancreas, gut, liver and exocrine glands (Ratjen et al., 2015). It is caused by mutations in the *CFTR* (Cystic Fibrosis Transmembrane Conductance Regulator) gene, which encodes a chloride channel involved in ion transport across epithelial surfaces. This genetic alteration promotes chronic infection, especially in the respiratory tract, due to thick and sticky mucus. In this context, mucus obstruction and progressive airway functional decline were detectable (Davis et al., 1996; Schögler et al., 2016; Stanford et al., 2021). Therefore, people with CF (pwCF) were initially presumed to be at high risk for severe COVID-19 outcomes (Fainardi et al., 2020). Contrary to expectations, several multinational cohort studies reported relatively low SARS-CoV-2 infection rates and milder clinical courses in pwCF compared to the general population (Colombo et al., 2021; Cosgriff et al., 2020; Mathew et al., 2021; McClenaghan et al., 2020). Recent *in vitro* findings further support this clinical observation, thus demonstrating that CFTR-deficient epithelial cells exhibit reduced susceptibility to SARS-CoV-2 infection, although the mechanisms remain elusive (Bezzerri et al., 2023a; Lagni et al., 2023; Lotti et al., 2022).

A hallmark of CF pathology is the disruption of intracellular chloride and bicarbonate transport due to mutations in the *CFTR* gene (Collawn and Matalon, 2014), which leads to hyperacidification of organelles, including the endoplasmic reticulum (ER), Golgi apparatus, and endo-lysosomal compartments (Chen et al., 2009; Walker et al., 2016). This altered pH environment affects numerous cellular processes, including protein folding, protein glycosylation, trafficking, and host-pathogen interactions. Given that SARS-CoV-2 relies on a functional secretory pathway (Ameen et al., 2006; Yoo et al., 2002) and proper organelle pH homeostasis for the glycosylation and maturation of its spike protein (Ehrlich et al., 2020; Liang et al., 2022), as well as for *ACE2* receptor processing, the intracellular acidification characteristic of CF cells may impair critical steps in the viral life cycle, ranging from entry and replication to assembly and egress (Badawi and Ali, 2021; Vickers et al., 2002). Moreover, CF is associated with defective autophagy, a pathway that the virus exploits to form double-membrane vesicles (DMVs) involved in the viral replication. Impaired autophagy may therefore hinder DMV formation and limit viral replication in CFTR-deficient cells (Ji et al., 2023; Luciani et al., 2010; Marat and Haucke, 2016).

In this study, we investigate the molecular basis of SARS-CoV-2 restriction in CF by conducting a transcriptomic analysis of wild-type and CFTR-mutant bronchial epithelial cells, both under baseline conditions and following viral infection. By investigating alterations in intracellular trafficking, organelle acidification, and autophagy-related pathways, we investigated the cellular mechanisms that may contribute to the reduced susceptibility to SARS-CoV-2 detected in pwCF.

## 2 Materials and methods

### 2.1 Cells

The CFBE41o^-^ cell line, immortalized with a pSVori plasmid, was employed. Cells stably expressing either wt-CFTR (WT) or F508del-CFTR (ΔF) were used (Bebok et al., 2005; Bruscia et al., 2002; Illek et al., 2008). Cells were maintained in Minimum Essential Medium (MEM, Gibco) supplemented with 10% Fetal Bovine Serum (FBS, Euroclone), 1% Glutamine (Gibco), and puromycin for selection.

To validate CFBE41o^-^ cell line results, the MucilAir™ model (Epithelix Sàrl), consisting of a fully differentiated 3D bronchial epithelium derived from primary human cells and cultured at an air–liquid interface (ALI), was employed. Models derived from healthy, non-smoking donors (MucilAir™ WT; n = 3, mean age 46 ± 16, sex: 1 male and 2 female, origin: one Caucasian and two African) and from non-smoking CF patients homozygous for ΔF508 (MucilAir™ F508del^+/+^; n = 3, mean age 28 ± 10, all female, origin not specified) were included. All donor-derived cultures displayed mucus production.

For MucilAir™ experiments, biological replicates were defined as independent donor-derived cultures (n=3 per condition), technical replicates (n=3) consisted of independent cultures derived from the same donor, which were separately infected and processed for all downstream analyses.

For MucilAir™ experiments, biological replicates were defined as independent donor-derived cultures (n = 3 per condition), while technical replicates (n = 3) consisted of independent cultures generated from the same donor, which were separately infected and processed for all downstream analyses.

### 2.2 SARS-CoV-2 infection and RNA extraction

The SARS-CoV-2 B.1 strain (hCoV-19/Italy/BO-VB12/2020|EPI_ISL_16978127) was used to inoculate cells at a multiplicity of infection (MOI) of 1 (Ogando et al., 2020). Cells were harvested before infection and at 24-, 48-, and 72-hours post-infection (hpi). Total intracellular RNA was extracted using the ReliaPrep RNA Cell Miniprep System (Promega, Madison, WI, USA). RNA quantity and integrity were assessed via Qubit Fluorometric Quantification (ThermoFisher, Waltham, MA, USA) and Fragment Analyzer system (Agilent, Santa Clara, CA, USA), respectively. Four independent biological experiments were performed using CFBE41o^-^ cells, and three independent biological experiments were performed using MucilAir™ cultures. Each biological experiment included three technical replicates.

### 2.3 RNA sequencing

Total RNA quality was assessed using the Agilent 2100 Bioanalyzer and samples with RIN > 6.0 were processed. Library preparation was performed using poly(A) selection and paired-end sequencing (2 × 150 bp) was carried out by Genewiz (Leipzig, Germany) on Illumina HiSeq (CFBE41o^−^) or NovaSeq (MucilAir™) platforms, generating 20–30 million reads per sample (≥80% bases with Q30). Reads were trimmed with Trimmomatic v0.36 to remove adapters and low-quality bases and aligned using STAR v2.5.2b to a combined reference genome (human GRCh38.p7, ENSEMBL release 87 + SARS-CoV-2 Wuhan-Hu-1 NC_045512.2). Only uniquely mapped reads were retained.

### 2.4 Bioinformatics and differential expression analysis

Gene-level quantification was performed using featureCounts (Subread v1.5.2) with ENSEMBL GRCh38.87 annotation. Differential expression analysis was conducted using DESeq2 v1.30.1. The Wald test was used to generate p-values and log2 fold changes. Genes with adjusted p-value < 0.05 and log2 fold change > 1 were considered differentially expressed. Functional enrichment analysis was performed using WebGestalt (WEB-based GEneSeTAnaLysis Toolkit) (Liao et al., 2019) using GO and KEGG databases through Over-Representation Analysis (ORA) and Gene Set Enrichment Analysis (GSEA). The Benjamini–Hochberg method was applied for multiple testing correction (FDR < 0.05).

### 2.5 RT-qPCR validation of RNA-seq data

SARS-CoV-2 RNA in culture supernatants was quantified using the multiplex real-time PCR Allplex™ 2019-nCoV assay (Seegene, Seoul, Republic of Korea), targeting the E, RdRp/S, and N genes, according to the manufacturer instructions.

RNA was retrotranscribed (Improm-IITM Reverse Transcriptase System, Promega) and qPCR was performed on a CFX96 Real-Time System (Bio-Rad, California, USA) using GoTaq^®^ qPCR Master Mix (Promega). Selected deregulated genes were validated using *GAPDH* as the reference gene. Primers are indicated in **Table 1**.

**Table 1:** Primers used for RT-qPCR.

Gene	Forward (5’→3’)	Reverse (5’→3’)
*OAS1*	AGGAAAGGTGCTTCCGAGGTAG	GGACTGAGGAAGACAACCAGGT
*OAS2*	GCTTCCGACAATCAACAGCCAAG	CTTGACGATTTTGTGCCGCTCG
*CASP1*	GCTGAGGTTGACATCACAGGCA	TGCTGTCAGAGGTCTTGTGCTC
*CALML5*	TGGAAACGGCACCATCAATGCC	ACTCCTGGAAGCTGATTTCGCC
*EGR3*	GACTCGGTAGTCCATTACAATCAG	AGTAGGTCACGGTCTTGTTGCC
*FOS*	GCCTCTCTTACTACCACTCACC	AGATGGCAGTGACCGTGGGAAT
*GAPDH*	TCAAGAAGGTGGTGAAGCAGG	CAGCGTCAAAGGTGGAGGAGT

Data are presented as the mean ± SD from three independent experiments in triplicate. Statistical analysis was performed using 2-way ANOVA in GraphPad Prism version 10 (GraphPad software Inc., La Jolla, CA, USA), with p < 0.05 considered significant.

## 3 Results

### 3.1 Baseline transcriptomic differences between CFBE41o^-^ ΔF and WT cells reflect altered intracellular environment and pH regulatory pathways

**Figure 1:**
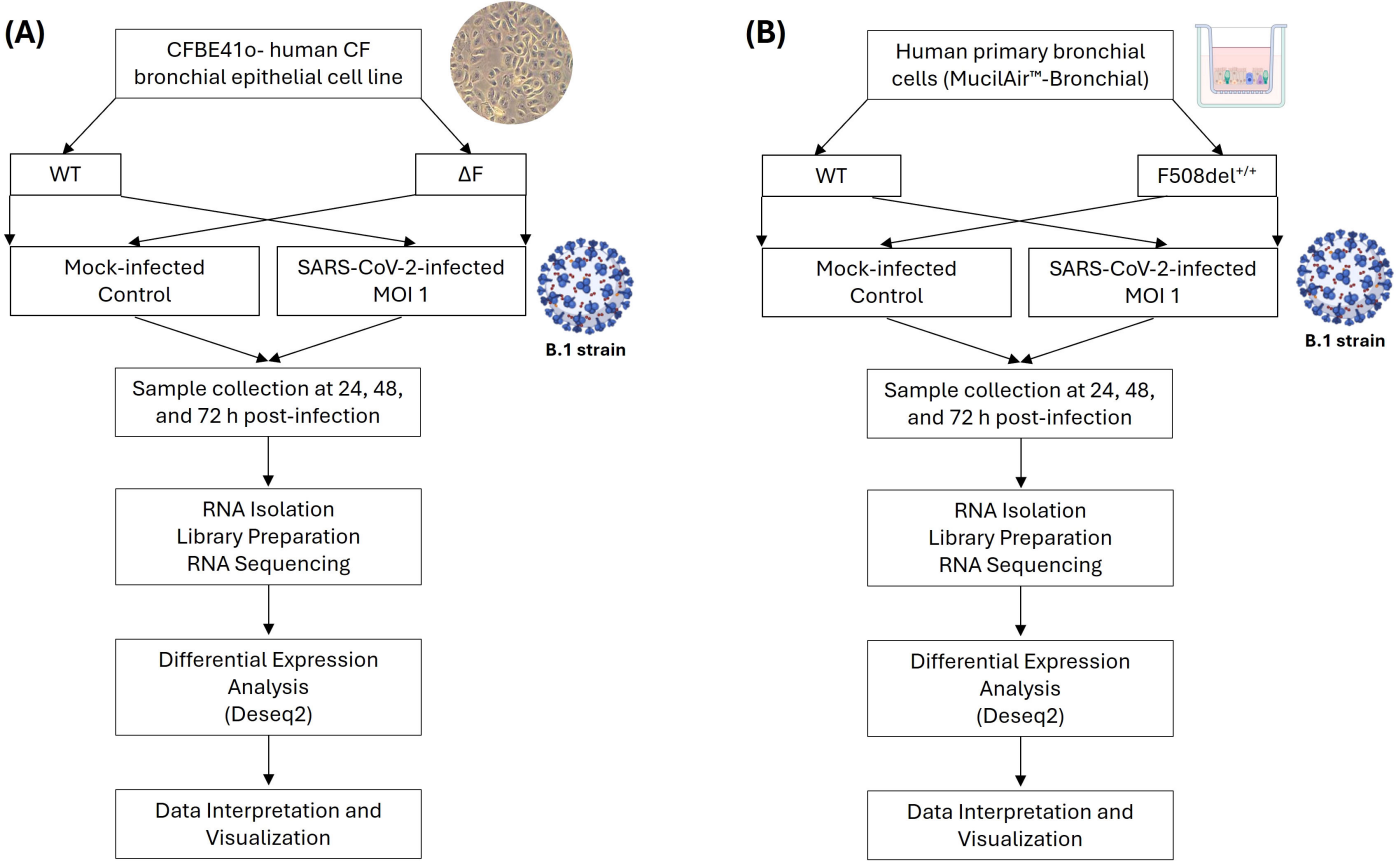
Schematic workflow describing experimental procedure and transcriptomic analysis. **(A)** CFBE41o^-^ stably expressing wt-CFTR (CFBE41o^-^ WT) and F508del-CFTR (CFBE41o^-^ ΔF) were utilized. CFBE41o^-^ were established for infection with SARS-CoV-2 B.1 strain at MOI 1. At 48 h post-infection, RNA was isolated and processed for library preparation. Sample libraries were sequenced on the Illumina HiSeq 2000 platform. Differentially expressed genes were identified and analyzed through Over-Representation Analysis (ORA) and Gene Set Enrichment Analysis (GSEA) to determine the over-represented or under-represented biological pathways. **(B)** Schematic workflow on WT and F508del^+/+^ MucilAir™.

In the first set of experiments, we evaluated the mRNA expression in CFBE41o^-^ ΔF cells (where the CFTR gene carries a deletion of the phenylalanine residue at position 508) and CFBE41o^-^ wild type (WT) cells (**Figure 1A**).

**Figure 2:**
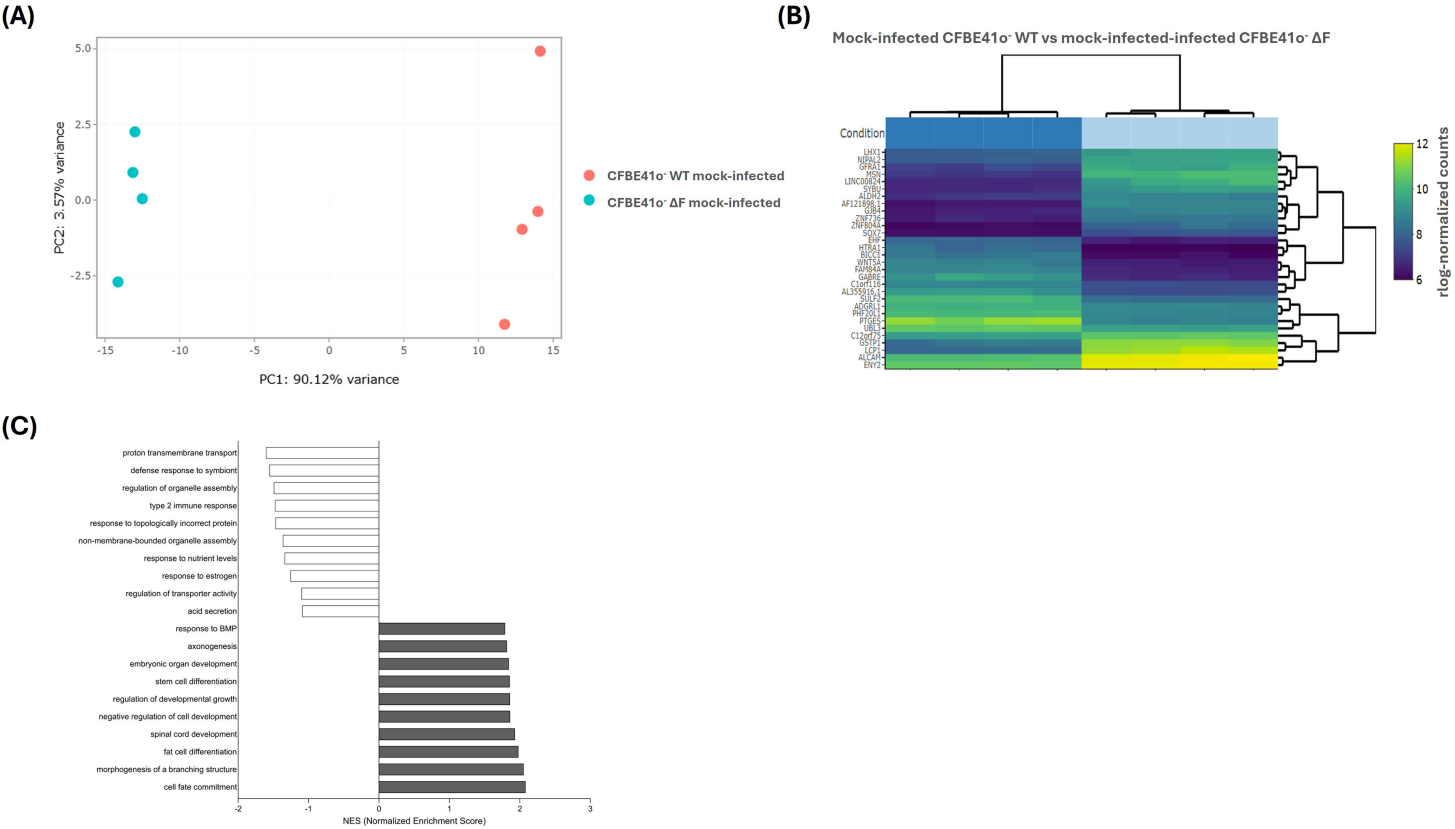
Transcriptomic alterations between mock-infected CFBE41o^-^ WT and ΔF cells. **(A)** Principal component analysis (PCA) of samples based on rlog-normalized gene expression values. Samples are projected onto the first two principal components, which explain the largest sources of variance in the dataset. **(B)** A bi-clustering heatmap visualizing the expression profile of the top 30 DEGs of mock-infected CFBE41o^-^ WT vs ΔF cells sorted by their adjusted p-value. Colors represent rlog-normalized expression values, where higher (yellow) and lower (purple) intensities correspond to relatively up- or down-regulated genes across the two conditions. **(C)** Gene Ontology Enriched gene set enrichment analysis (GSEA). In grey, the terms with positive normalized enriched score (NES), whereas in white the terms with negative NES.

To determine whether the CFBE41o^-^ ΔF cells transcriptional profiles are intrinsically different from WT cells, mock-infected baseline CFBE41o^-^ WT and ΔF were analyzed for differential gene expression (**Figures 2A, B**) by RNA-seq procedure.

At baseline, we observed 2125 DEGs between mock-infected ΔF and WT CFBE41o^-^ cells (781 downregulated, 1344 upregulated in ΔF). GO analysis showed that response to nutrient levels, cellular response to biotic stimulus, type 2 immune response, regulation of organelle assembly, defense response to symbiont, and response to virus were downregulated in ΔF cells, suggesting a downregulation of immune, metabolic, and environmental response pathways (**Figure 2C**).

At baseline, CFBE41o^-^ ΔF showed a transcriptional profile consistent with heightened innate immune activation. Key signaling components such as SYK, CD14, and CD11b were upregulated, together with inflammatory mediators including IL17D, CCDC88B, and SCARA3, indicating enhanced readiness for leukocyte recruitment and inflammatory amplification. Several interferon-stimulated genes (IFITM2, IFITM10, IFI16, IFI44) were also induced, pointing to higher immune sensing. In contrast, classical antiviral restriction factors such as SLFN5, MLKL, SAMD9L, and APOBEC3B were suppressed, suggesting that CF cells combine exaggerated pro-inflammatory priming with reduced antiviral competence.

Top DEGs (**Figure 2B**; **Table 2**) included developmental regulators (*SOX1, WT1, ZNF135*), which point to dedifferentiation and reactivation of morphogenetic pathways silenced in mature airway epithelium (Kreidberg, 2010; Venere et al., 2012), and genes for ion transport and pH regulation (*SLC4A4*).

**Table 2:** Top deregulated genes for the most relevant functional category in mock-infected CFBE41o^-^ ΔF cells respect to CFBE41o^-^ WT.

Functional category	Gene	Function	Log2FC
Adhesion	*CDH13*	Atypical cadherin; modulates cell adhesion and signaling	-7,815265
Autophagy	*MAP1LC3A*	Microtubule-associated protein 1 light chain 3 alpha	-1.7311
	*TFEB*	Transcription Factor EB	1.9788
	*USP13*	Ubiquitin-specific peptidase 13	-1.0983
	*SQLE*	Squalene epoxidase	-1.1215
Senescence	*CDKN1A*	Cyclin Dependent Kinase Inhibitor 1A (p21)	1.4721
	*PTGS2*	Prostaglandin-Endoperoxide Synthase 2 (COX-2)	2.7802
	*MMP9*	Matrix Metallopeptidase 9	-3.3913
	*IL6R*	Interleukin 6 Receptor	1.4705
	*DUSP1*	Dual Specificity Phosphatase 1	1.7848
	*IGFBP5*	Insulin-like Growth Factor Binding Protein 5	4.5874
Cytoskeleton	*MSN*	Membrane-cytoskeleton linker protein; structural integrity	-6,45504
Cytoskeleton/signaling	*EPHA4*	Ephrin receptor; cell architecture and signal transduction	1,7378148
	*EPHB1*	Ephrin receptor; modulates cytoskeletal dynamics and intracellular signaling	2,6390818
	*EPHB3*	Ephrin receptor; involved in organelle trafficking and remodeling	1,0080119
	*EPHB6*	Ephrin receptor; cytoskeletal rearrangement and morphogenesis	1,7282556
	*PHACTR3*	Actin regulator; modulates cytoskeleton and phosphatase activity	1,692313
Developmental regulation	*EYA2*	Cofactor in developmental signaling and organogenesis	5,6154076
	*SOX1*	Transcription factor regulating neural and epithelial fate	6,5038101
	*WT1*	Key transcriptional regulator of development and cell survival	5,6982634
	*ZNF135*	Zinc finger protein involved in transcriptional repression and chromatin remodeling	-5,623648
ER function/pH regulation	*ASPH*	ER-localized hydroxylase; involved in calcium homeostasis and protein modification	-1,204045
Ion transport	*GABRE*	GABA-A receptor subunit; modulates chloride ion transport	5,583859
	*SLC4A4*	Sodium bicarbonate transporter; regulates intracellular pH	6,6758604
Metabolism	*PID1*	Modulates insulin signaling and mitochondrial metabolism	5,5724741
Lipid metabolism	*UGT8*	UDP-galactose ceramide galactosyltransferase; involved in myelination/lipid metabolism	5,7876219
Mitochondrial organization	*SNPH*	Mitochondrial anchor protein; regulates organelle positioning and transport	1,316564
Transport	*SLCO1B3*	Organic anion transporter; involved in bile acid and drug transport	6,0281557
Vesicle trafficking	*RPH3AL*	Vesicle exocytosis regulator; linked to synaptic-like secretory pathways	-6,98074
pH regulation/Amino acid transport	*SLC7A14*	Solute carrier family member; regulates lysosomal amino acid homeostasis and intracellular pH	-7,576354

Downregulation of the ER-localized, pH-sensitive hydroxylase *ASPH* suggested defects in calcium signaling and protein maturation, key processes in the airway antimicrobial defense system. *ASPH* is most active at neutral to slightly alkaline pH (Brewitz et al., 2020). Cellular pH affects *ASPH*-related processes like cell migration and epithelial-to-mesenchymal transition (EMT) (Zou et al., 2018).

Alterations of these signatures can impair vesicle maturation, endosomal trafficking, and mucosal hydration, suggesting a deep dysregulation of intracellular and mucosal functional regulation. A hallmark of this derangement is related to the differential mRNA expression in genes involved in vesicular trafficking and membrane organization, such as *RPH3AL*, a Rab effector promoting vesicle exocytosis (Martinez-Arroyo et al., 2021), and ephrin receptor kinases *EPHB1, EPHB3, EPHB6*, and *EPHA4* (Stallaert et al., 2018), which orchestrate cytoskeletal remodeling and intracellular signaling. The actin regulator *PHACTR3* and mitochondrial anchor *SNPH* further implicate dysfunction in cytoskeleton-mitochondria coordination, essential for organelle positioning and energy distribution (Caino et al., 2017; Itoh et al., 2014).

We also observed deregulation of glycosylation enzymes (GALNT14, GALNT13, HS6ST2), consistent with the altered glycosylation pattern reported in CF airways, affecting also mucin processing and extracellular matrix remodeling (Scanlin and Glick, 2001; Schulz et al., 2007), as well as cytoskeletal regulators (*MSN*) reflecting chronic cytoskeletal remodeling that can interfere with vesicle mobility and apical organization (Abiatari et al., 2010).

Transporters such as *SLCO1B3* and metabolic enzymes like *PID1* and *UGT8* were also differentially expressed, indicating shifts in solute handling and mitochondrial stress adaptation (Hao et al., 2023; Marin et al., 2024; Zhang et al., 2024).

The analysis of RNA-seq results also displayed a differential expression in several genes involved in autophagy and senescence regulation. Interestingly, different genes involved in autophagy were dysregulated in ΔF cells; *MAP1LC3A, USP13, CHMP4B* and *SQLE* were downregulated, suggesting a potential reduction in autophagosome formation and autophagic flux (Zhen et al., 2020).

Senescence-associated genes (*CDKN1A, IGFBP5, DUSP1*) were upregulated, indicating a pro-senescent phenotype (Alessio et al., 2024; Cheng et al., 2018; López-Domínguez et al., 2021). Similarly, *PTGS2* was also upregulated, supporting the presence of a pro-inflammatory senescence-associated secretory phenotype (SASP) in ΔF cells (Gonçalves et al., 2021). *MMP9* was downregulated in ΔF cells, suggesting the onset of premature senescence (Rao et al., 2007).

In addition to broad alterations in immune and intracellular homeostatic pathways, analysis of cilia-associated transcripts revealed baseline modulation of genes involved in ciliary structure and assembly in CFBE41o^-^ ΔF cells. Several axonemal and intraflagellar transport components (IFT88, DNAI1, DNAH11, CCDC40, CFAP74), indicating gene-specific deregulation of cilia-related transcripts under baseline conditions. Importantly, these changes did not indicate activation of a coordinated ciliogenesis program but rather a heterogeneous modulation of structural ciliary components, consistent with altered epithelial organization associated with CFTR dysfunction.

Together, these transcriptional changes suggest a strong altered state in epithelial homeostasis in CFBE41o^-^ ΔF cells, characterized by disorganized intracellular architecture, impaired immune readiness, and altered intracellular acidification.

### 3.2 Transcriptomic profiling of SARS-CoV-2-infected bronchial epithelial cells reveals a major direct virus-host interaction at 48 hours post-infection

**Figure 3:**
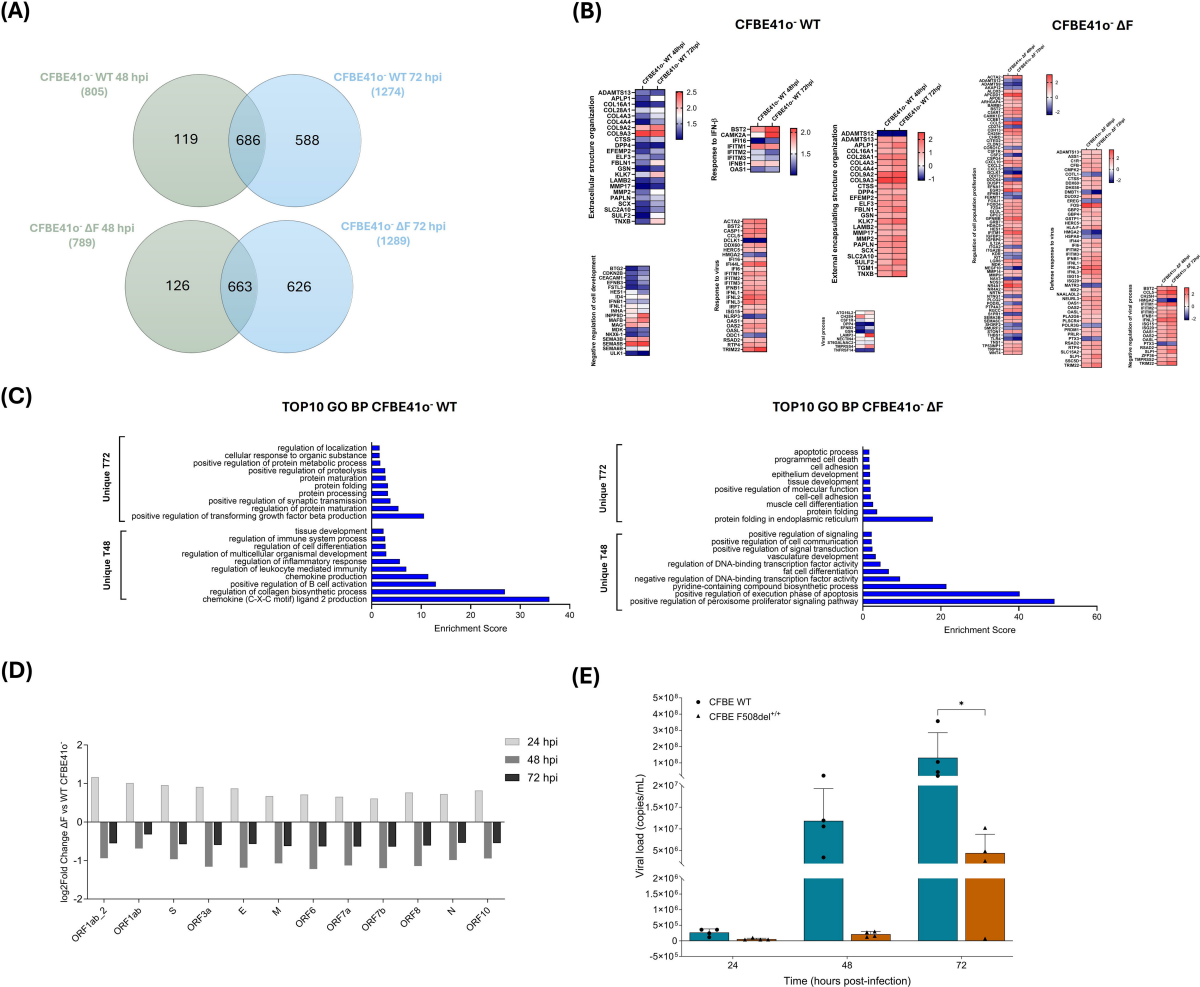
DEGs in response to SARS-CoV-2 infection in CFBE41o^-^ WT and ΔF cells at 48 and 72 hpi. **(A)** Venn diagrams illustrate the overlap of DEGs between 48 and 72 hpi in CFBE41o^-^ WT (top) and in CFBE41o^-^ ΔF (bottom) cells. **(B)** Heatmaps with expression profiles of DEGs common to both time points for WT and ΔF cells, divided by their function. **(C)** Bar charts reporting the top 10 enriched GO terms for genes specifically regulated in CFBE41o^-^ WT (left) and CFBE41o^-^ ΔF (right) cells. **(D)** Bar plots with the log_2_ fold change of viral gene expression at 24, 48, and 72 hpi in CFBE41o^-^ ΔF vs WT cells. **(E)** Quantification of SARS-CoV-2 viral load in culture supernatants from CFBE41o^-^ WT and F508del^+/+^ cells at 24, 48, and 72 hpi, measured by multiplex real-time PCR (Seegene). The data are presented as the mean ± SD from independent experiments (n = 4; *p < 0.05).

In the next series of experiments, we carried out RNA-seq analysis in the previously described cell lines infected with SARS-CoV-2 to investigate the viral impact in these cellular contexts. To characterize the temporal dynamics of the host response to SARS-CoV-2 infection, we performed transcriptomic analyses of CFBE41o^-^ models at 24, 48, and 72 hpi (**Figure 3A**). Transcriptomic profiling of SARS-CoV-2-infected WT and ΔF CFBE41o^-^ cells revealed peak host gene deregulation at 48 hpi, sustained but more heterogeneous by 72 hpi, with no-statistically significant changes at 24 hpi. At 48 hpi, both cell models showed deregulation of interferon-stimulated genes, chemokines, pro-apoptotic markers, and activation of antiviral pathways, including interferon signaling, immune activation, cellular stress responses, and autophagy. By 72 hpi, transcriptional profiles shifted toward homeostatic and developmental programs (**Figures 3B, C**).

We also assessed the time-dependent expression dynamics of viral genes in CFBE41o^-^ ΔF vs WT cells (**Figure 3D**). We observed that viral expression levels were higher at 24 hpi in CFBE41o^-^ ΔF cells compared to WT cells, suggesting more efficient viral entry and/or in the early development of infection. However, viral gene expression markedly decreased in ΔF cells at 48 hpi while it increased in WT cells, a trend that persists at 72 hpi.

To complement intracellular viral gene expression analyses, SARS-CoV-2 RNA levels were quantified in culture supernatants at 24, 48, and 72 hpi (**Figure 3E**). At 24 hpi, viral RNA levels were low and close to background in both CFBE41o^-^ WT and F508del^+/+^ cells. By 48 hpi, WT cells exhibited an increase in viral RNA detected in the supernatant, whereas CFTR-deficient cells showed persistently low viral loads. At 72 hpi, WT cultures continued to exhibit increasing viral RNA levels in the supernatant, while F508del^+/+^ cells showed a limited increase and maintained markedly reduced viral loads compared to WT.

In combination with the intracellular viral gene expression profiles, these results indicate that, although early phases of infection can be initiated in CFTR-deficient cells, sustained productive viral replication and/or release becomes progressively impaired at later stages, consistent with the establishment of a non-permissive intracellular environment as already suggested in our previous work (Lagni et al., 2023).

Altogether, these results establish the infection time of 48 hours as the optimal window for studying the direct transcriptional impact of SARS-CoV-2 in airway epithelia, capturing peak antiviral activity before later cell-intrinsic and tissue remodeling mechanisms emerge. In line with other studies (Blanco-Melo et al., 2020; Ravindra et al., 2021; Wyler et al., 2021), we focused our additional analyses on this time point.

### 3.3 SARS-CoV-2 induces extensive transcriptional remodeling in CFBE41o^-^ WT cells targeting immune regulation, ion transport and intracellular organization

**Figure 4:**
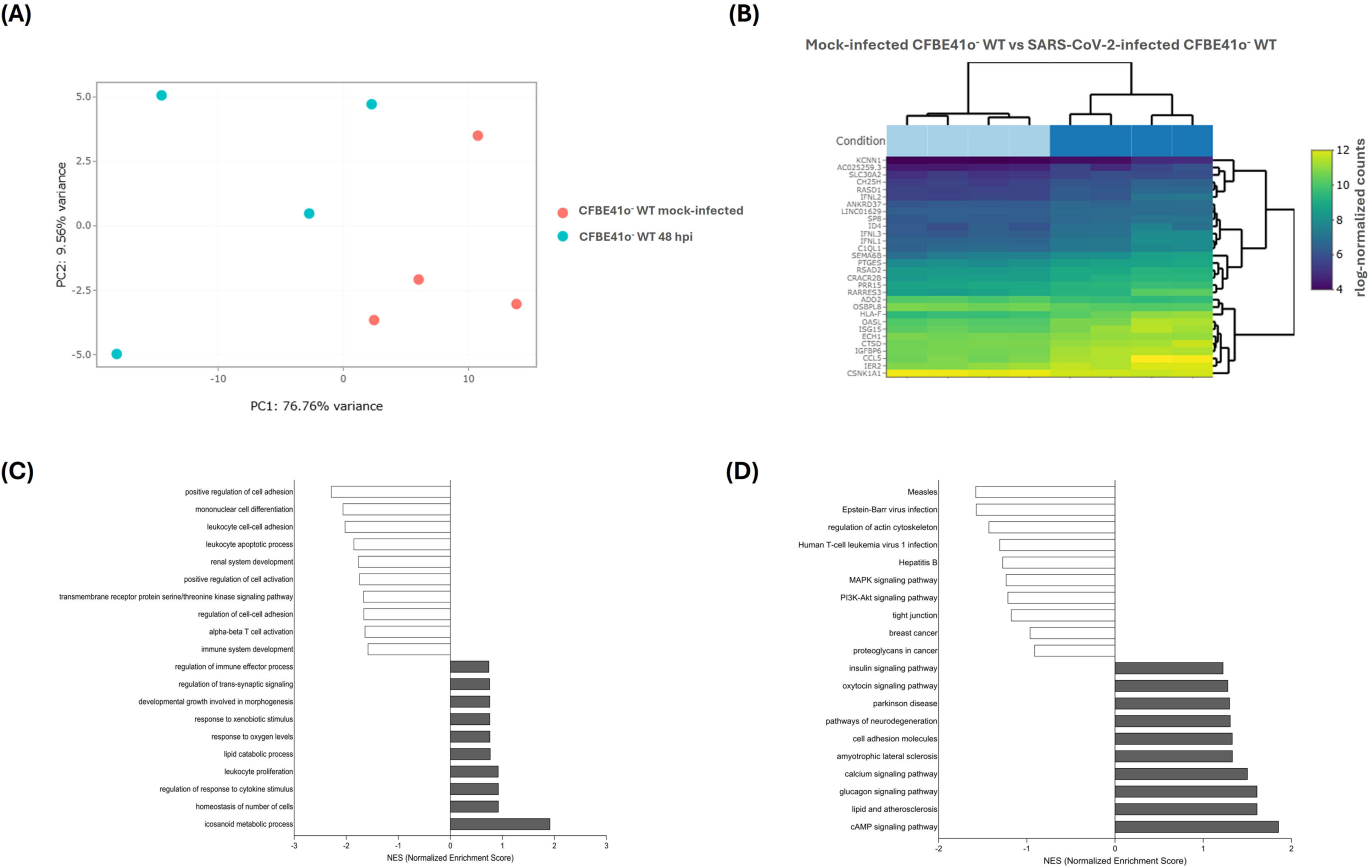
Transcriptional response and pathway enrichment in SARS-CoV-2-infected CFBE41o^-^ WT cells. **(A)** Principal component analysis (PCA) of samples based on rlog-normalized gene expression values. Samples are projected onto the first two principal components, which explain the largest sources of variance in the dataset. **(B)** Bi-clustering heatmap of the top 30 differentially expressed genes (DEGs) ranked by adjusted p-value, illustrating expression profiles across samples. Colors represent rlog-normalized expression values, where higher (yellow) and lower (purple) intensities correspond to relatively up- or down-regulated genes across the two conditions. **(C)** GO Biological Process enrichment analysis of unique DEGs in SARS-CoV-2-infected vs mock-infected CFBE41o^-^ WT cells. **(D)** KEGG pathway enrichment analysis of the same DEGs, displaying normalized enrichment scores (NES) for the top enriched (grey bars) and depleted (white bars) pathways.

Analysis of response profiles in viral infected CFBE41o^-^ WT cells indicates that SARS-CoV-2 differentially modulated the expression of several genes at 48 hpi, inducing 805 DEGs (724 up, 93 down) (**Figure 4**).

The response included strong upregulation of innate immune and inflammatory genes (IL6, TGFB1, NFKBIZ, BCL6, JAK3), typical of an early epithelial viral response (Blanco-Melo et al., 2020; Ravindra et al., 2021). In contrast, genes governing adaptive immunity, such as those involved in T cell activation, leukocyte adhesion, and lymphocyte differentiation, were notably downregulated, suggesting an unbalanced immune response and impaired recruitment or activation of immune cells, features also observed in severe COVID-19 (Lucas et al., 2020).

SARS-CoV-2 infection induced significant changes in intracellular organization and stress adaptation. Notably, we observed upregulation of *ATP6V1B1*, a subunit of the vacuolar ATPase (V-ATPase) complex, along with ion transporters *KCNN1*, *GABRP*, and *TRPV6*, implicating a shift toward endolysosomal acidification that may promote viral entry (Bestle et al., 2020; Ikeuchi et al., 2024). In particular, elevated *ATP6V1B1* expression has been associated with lysosomal hyperacidification, a feature the virus may exploit to enhance its replication environment.

SARS-CoV-2 infection also promoted changes in cytoskeletal architecture, with increased transcription of *PRPH* and *TPPP3*, indicating reorganization of intermediate filaments and microtubule-associated proteins likely promoting intracellular viral transport and virion assembly (Lim et al., 2021; Öhman et al., 2009).

A hallmark of the host response was upregulation of the AP-1 transcription factor complex (*JUNB, FOS, FOSB*), which drives stress-induced reprogramming, in line with other studies showing AP-1 activation as a downstream consequence of SARS-CoV-2-mediated MAPK and oxidative stress pathways (Bouhaddou et al., 2020; Jiang et al., 2022). AP-1 induction also links to a senescence-like phenotype, with upregulation of *CDKN1A, CDKN2B*, and *IL6*, key SASP components, suggesting that viral infection not only arrests cell proliferation but also promotes a chronic inflammatory state, consistent with senescence induction detected in epithelial and endothelial models of SARS-CoV-2 infection (Lee et al., 2021).

Additionally, a robust autophagy-related program was triggered, with an upregulation of several core autophagy genes (*MAP1LC3A, ATG9B, ATG16L2, LAMP3*) and stress-responsive autophagy pathway genes (*TP53INP1/2, FOXO1/4*), suggesting enhanced autophagosome formation and lysosomal fusion. These findings align with previous reports indicating that SARS-CoV-2 can manipulate autophagic flux, both as a mechanism of immune evasion and to facilitate replication (Gassen et al., 2021; Miao et al., 2021).

In summary, in CFBE41o^-^ WT cells, SARS-CoV-2 infection triggers a targeted response (**Table 3**) involving immune activation, intracellular acidification, AP-1 transcription, and autophagy/senescence pathways. These changes reflect both antiviral defense and potential viral subversion, reshaping epithelial function through disrupted ion transport, immune signaling, and stress-related pathways.

**Table 3:** Top 20 DEGs divided by their function in SARS-CoV-2-infected respect to mock-infected CFBE41o^-^ WT cells.

Functional category	Gene	Notes	log2FC
Transcriptional regulation & immediate early response	*TCIM*	Wnt/β-catenin signaling coactivator	4.51
	*FOSB*	Immediate early gene; inflammation/stress response	3.91
	*FOS*	Immediate early gene; stress, inflammation	3.38
	*EGR2*	Transcription factor; immune & neuronal signaling	3.67
	*LMO1*	Transcriptional regulator; development	3.78
	*NR4A1*	Nuclear receptor; immune response, metabolic regulation	3.21
	*SAMD11*	Putative transcription regulator; apoptosis-related	3.25
Ion transport & membrane potential	*GABRP*	GABA-A receptor subunit; ion transport	4.11
	*KCNN1*	Calcium-activated K+ channel; membrane potential regulation	4.05
	*TRPV6*	Calcium-selective ion channel	3.16
	*ATP6V1B1*	V-ATPase subunit; lysosomal acidification	3.76
Cell survival, apoptosis, immune modulation	*BIRC7*	Inhibitor of apoptosis protein (IAP); cell survival	3.46
	*VTCN1*	B7-H4 immune checkpoint; T cell inhibition	3.79
Cytoskeletal & structural proteins	*PRPH*	Intermediate filament protein; cell structure	3.79
	*TPPP3*	Tubulin polymerization protein; cytoskeletal stability	3.40
Metabolism, redox, and enzymatic activity	*AKR1B15*	Aldo-keto reductase; detoxification, redox processes	3.82
	*CPXM1*	Carboxypeptidase-like; extracellular matrix remodeling	3.23
Immune/antiviral cytokine	*IFNL1*	Type III interferon; antiviral defense	2.82
	*IFNL2*	Type III interferon; antiviral defense at mucosal surfaces	2.82
	*IFNL3*	Type III interferon; similar to *IFNL2*	2.45

### 3.4 SARS-CoV-2 induces transcriptional remodeling in CFBE41o^-^ ΔF cells without perturbing autophagy, ER stress, or intracellular organization

**Figure 5:**
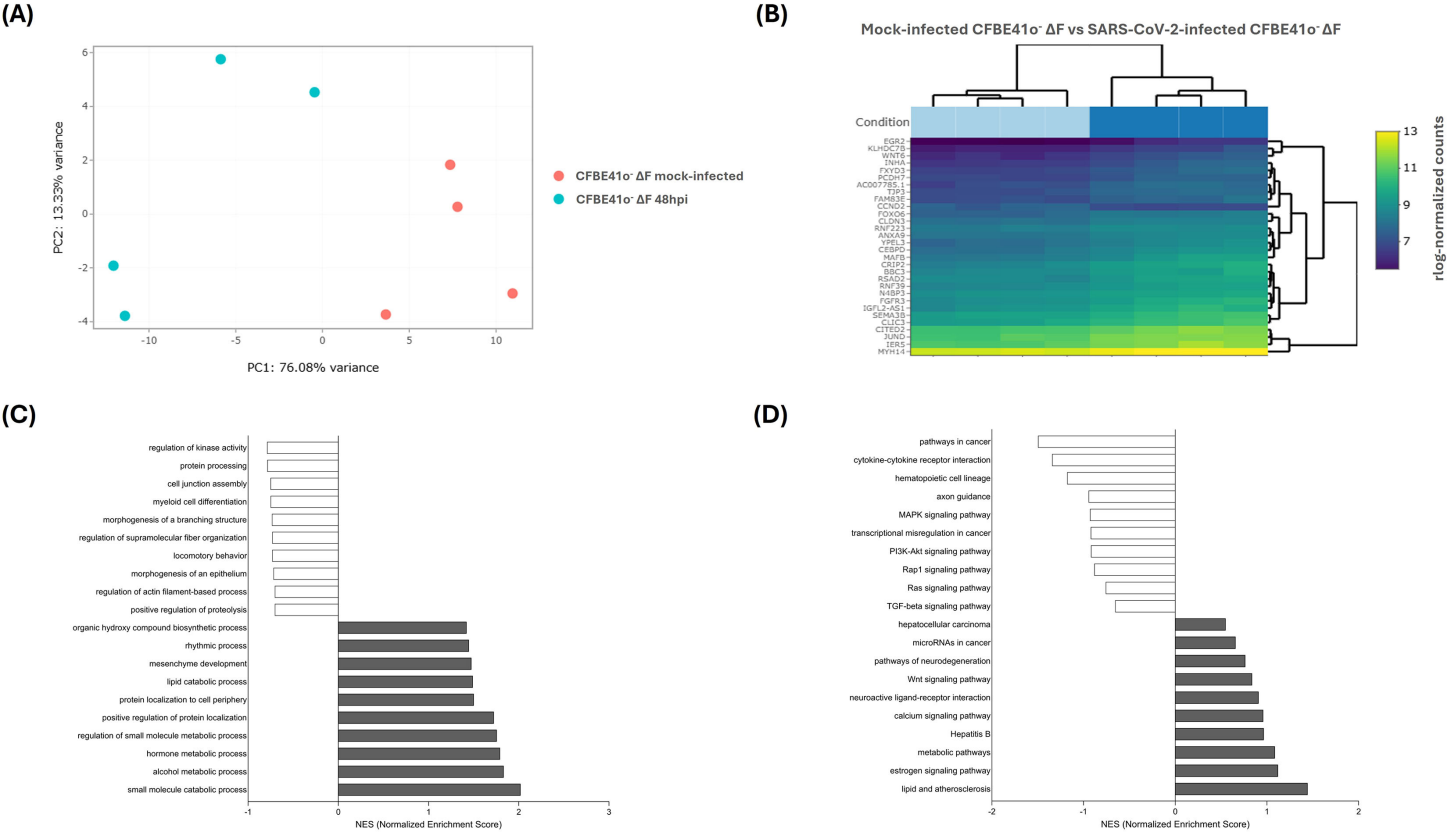
Transcriptional response and pathway enrichment in SARS-CoV-2-infected CFBE41o^-^ ΔF cells. **(A)** Principal component analysis (PCA) of samples based on rlog-normalized gene expression values. Samples are projected onto the first two principal components, which explain the largest sources of variance in the dataset. **(B)** Bi-clustering heatmap of the top 30 differentially expressed genes (DEGs) ranked by adjusted p-value, illustrating expression profiles across samples. Colors represent rlog-normalized expression values, where higher (yellow) and lower (purple) intensities correspond to relatively up- or down-regulated genes across the two conditions. **(C)** GO Biological Process enrichment analysis of unique DEGs in SARS-CoV-2-infected vs mock-infected CFBE41o^-^ ΔF cells. **(D)** KEGG pathway enrichment analysis of the same DEGs, displaying normalized enrichment scores (NES) for the top enriched (grey bars) and depleted (white bars) pathways.

We next analyzed the RNAseq data to assess the transcriptomic response CFBE41o^-^ ΔF cells after 48 hours post SARS-CoV-2 infection. A total of 789 (620 upregulated, 169 downregulated) DEGs were observed in the CFBE41o^-^ ΔF cells response to SARS-CoV-2 (**Figure 5**).

In CFBE41o^-^ ΔF bronchial epithelial cells, SARS-CoV-2 infection triggered a limited transcriptional response marked by modest innate immune activation and early stress signaling (**Figures 5C, D**). Although type III interferons (*IFNL2, IFNL3*), CCL5, and antiviral effectors such as IFITM1 were upregulated, the IL6 signaling axis (*IL6R, SERPINB1, SMAD3, MUC5AC, LRG1, ATP6V1C2*, and *S100A9* genes), a core component of the early inflammatory response, was suppressed. This muted immune profile contrasts with the robust cytokine response typically observed in WT epithelial cells and in COVID-19 airway models (Oliva et al., 2025).

Notably, the virus still triggered upregulation of early transcription factors, including *JUN, ATF3, BATF EGR2*, and *NR4A1*, components of the AP-1 complex, even though with a lower magnitude respect with WT cells. Still this early stress signal appeared uncoupled from key downstream reprogramming events like autophagy, senescence, or pH remodeling, highlighting the functional constraints in the CF cellular environment.

A defining feature was the absence of transcriptional remodeling of lysosomal acidification pathways. Unlike SARS-CoV-2-infected WT cells, ΔF cells showed no significant changes in this pathway, likely reflecting the pre-existing hyper-acidified state of intracellular compartments. This baseline state may also explain the failure to activate autophagy-related genes, which were strongly induced in SARS-CoV-2-infected WT cells.

The absence of autophagy activation suggests that the virus cannot exploit a defective system. Similarly, key senescence and unfolded protein response genes remained unchanged, possibly because these stress pathways are already constitutively engaged in CF (Ribeiro and Boucher, 2010), and SARS-CoV-2 may be unable to further amplify these constitutively engaged or dysfunctional pathways.

Altogether, these findings indicate that pre-existing intracellular acidification and impaired homeostatic flexibility in CFBE41o^-^ ΔF cells blunt the transcriptional remodeling typically induced by SARS-CoV-2, limiting viral manipulation of host defense, vesicle trafficking, and organelle function. This restricted response may contribute to altered viral dynamics and epithelial pathophysiology in CF airways (**Table 4**).

**Table 4:** Top 20 DEGs divided by their function in SARS-CoV-2-infected CFBE41o^-^ ΔF cells with respect to mock-infected cells.

Functional category	Gene	Notes	log_2_FC
Cell adhesion	*VIT*	Extracellular matrix protein; involved in adhesion and signaling	4.08
Cytoskeleton/muscle	*MYOZ1*	Z-disc protein; regulates muscle contraction and structure	2.88
Immediate early gene/transcription factor	*FOSB*	AP-1 family; marker of acute cellular response	4.02
	*FOS*	AP-1 transcription factor; rapidly induced by stress	3.52
	*EGR2*	Regulates cell differentiation and growth	3.22
	*NR4A1*	Nuclear receptor; modulates inflammation and apoptosis	3.02
	*EGR3*	Involved in immune regulation and stress response	2.75
Immune/antiviral cytokine	*IFNL2*	Type III interferon; antiviral defense at mucosal surfaces	2.42
	*IFNL3*	Type III interferon; similar to *IFNL2*	2.45
Immune/antiviral defense	*IFITM1*	Blocks viral entry; membrane-associated protein	1.36
Immune/chemokine	*CCL5*	Recruits T and NK cells; involved in antiviral response	2.40
Immune/checkpoint	*VTCN1*	Inhibits T cell activation; immune regulatory role	2.74
Ion transport	*GABRP*	GABA-A receptor subunit; chloride ion transporter	3.37
Long non-coding RNA	*LINC02518*	Regulator of gene expression; function not well characterized	3.28
Metabolism/glucose	*SLC2A14*	Facilitates glucose uptake; insulin-independent transporter	2.35
Metabolism/hormone	*SULT1E1*	Sulfotransferase involved in estrogen metabolism	3.56
Non-coding RNA	*AC025259.3*	Putative non-coding RNA; function unknown	3.73
Pseudogene	*HCG4P11*	Pseudogene; unclear biological function	2.37
Wnt signaling	*APCDD1*	Wnt pathway inhibitor; regulates proliferation and differentiation	2.75
	*WNT6*	Role in the early development of embryos	2,33
Wnt signaling/growth	*WISP2*	Wnt target gene; involved in cell growth and tissue repair	2.59

### 3.5 Transcriptomic interaction analysis reveals an attenuated regulation of host pathways required for SARS-CoV-2 replication in CFBE41o^-^ ΔF cells compared to CFBE41o^-^ WT cells

To understand how SARS-CoV-2 affects CFBE41o^-^ ΔF cells differently from CFBE41o^-^ WT cells, an interaction analysis, which compares how gene expression changes after infection in each cell type, was performed. Each cell response to infection was calculated relative to its own baseline, and these changes were then compared across the two cell types. This approach highlights genes and pathways that are specifically altered by SARS-CoV-2 in a cell-dependent way, revealing how CFTR dysfunction impacts the viral infection response.

Transcriptomic analysis of CFBE41o^-^ WT and ΔF cells revealed at 48 hpi both shared and divergent transcriptional programs, emphasizing the functional consequences of CFTR dysfunction on host antiviral defenses and potential viral replication dynamics.

**Figure 6:**
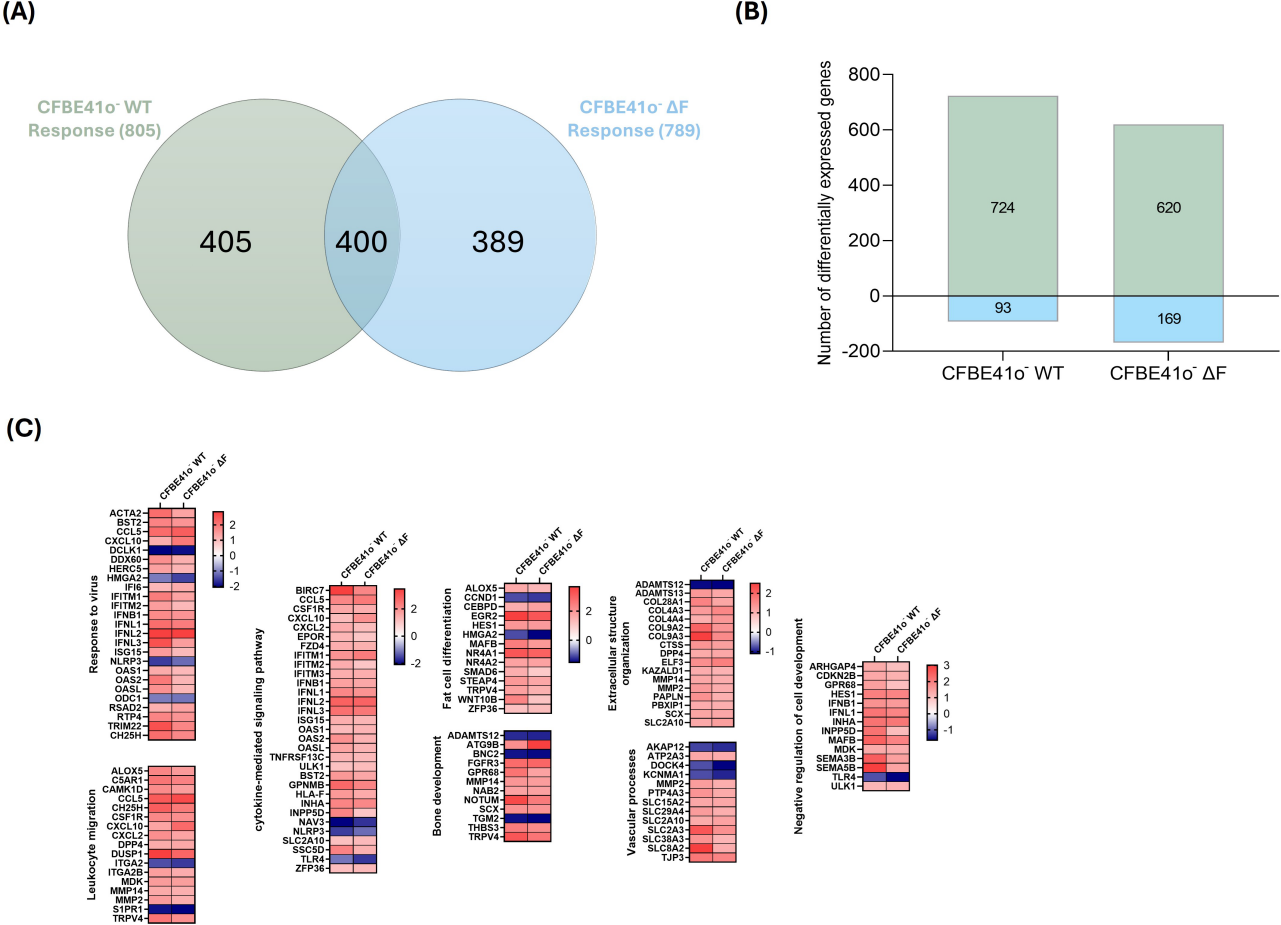
Comparison of SARS-CoV-2-induced transcriptional responses in CFBE41o^-^ WT and ΔF cells. **(A)** Venn diagram comparing the differentially expressed genes (DEGs) between CFBE41o^-^ WT cells (green; WT SARS-CoV-2 infected vs WT mock-infected cells) and CFBE41o^-^ ΔF cells (blue; ΔF SARS-CoV-2-infected vs ΔF mock-infected cells) response to SARS-CoV-2 infection. A total of 400 DEGs were common between WT and ΔF response to viral infection. **(B)** The number (y-axis) and direction of change (upregulated = positive y-axis, downregulated = negative y-axis) of DEGs (|Log_2_FC|1.0, adjusted p-value < 0.05) of WT and ΔF CFBE41o^-^ cells response to SARS-CoV-2 infection (x-axis). **(C)** The relative expression genes that are commonly differentially expressed (Log_2_FoldChange>1.0, adjusted p-value < 0.05) in CFBE41o^-^ WT and ΔF cells after SARS-CoV-2 infection.

A total of 789 DEGs were identified in the ΔF cells response, and 805 in the WT cells response. While there was considerable overlap (400 shared DEGs), WT cells exhibited a slightly higher number of unique DEGs (405) compared to ΔF (389) (**Figure 6**).

Analysis revealed both shared and genotype-specific responses to SARS-CoV-2 in CFBE41o^-^ WT and ΔF cells, with CFTR dysfunction dampening several key antiviral and stress-related pathways. CFBE41o^-^ ΔF cells exhibited reduced activation of endolysosomal acidification genes, including *ATP6V1B1*, reflecting defective vesicular acidification known to occur in CF (Barasch et al., 1991; Haggie and Verkman, 2009). This likely impairs viral uncoating, antigen presentation, and lysosomal degradation. Genes involved in vesicular trafficking and cytoskeletal remodeling (*MYOZ1, PRPH*) were also more strongly induced in CFBE41o^-^ WT cells, suggesting compromised intracellular transport and viral trafficking in the CF context. *KCNN1*, a Ca²^+^-activated potassium channel affecting vesicle fusion and inflammasome signaling, was among the most upregulated genes in WT but showed limited induction in ΔF cells, further implicating disrupted ion-dependent pathways.

The AP-1 transcription factor complex (*JUN, FOS, ATF3*) showed enhanced activation in WT cells, consistent with stronger engagement of MAPK signaling and cellular stress responses. This was paralleled by a more robust proinflammatory response in WT, with higher expression of interferons (*IFNB1, IFNL1-3*), Interferon stimulating genes (ISGs) (*IFITM1-3, OAS1/2, ISG15*), and chemokines (*CXCL10, CCL5, CXCL2*), indicating more effective viral sensing and immune priming. In contrast, ΔF cells mounted a weaker interferon response and showed reduced induction of upstream immune sensors (*DDX60, TLR4, UBA7*), suggesting attenuated innate immune activation. Importantly, autophagy-related (*AMBRA1, ATG9B*), senescence-associated genes, including SASP components and regulators (*CXCL10, ANG, EREG, HGFAC*), were also dampened in ΔF cells, pointing to impaired antiviral reprogramming.

Furthermore, ΔF cells showed weaker induction of endoplasmic reticulum (ER) stress and unfolded protein response (UPR) genes (*HSPA5, ATF4, DDIT3*), which are typically hijacked by SARS-CoV-2 for replication and protein folding.

Cilia-related genes were also analyzed to determine whether SARS-CoV-2 triggers genotype-dependent changes in ciliary differentiation or axonemal organization. Infected CFTR-deficient cells showed upregulation of FOXJ1 and DNAH7 together with strong downregulation of the dynein-arm assembly/trafficking factor DAW1, whereas infected WT cells preferentially upregulated DNAH1 and the ciliated/epithelial differentiation marker *TPPP3*. ZMYND10 and LRRC56 were induced in both conditions. Overall, these changes reflect gene-specific modulation rather than a coordinated, directional program, indicating the absence of a coordinated activation of broad ciliogenesis or axonemal remodeling programs in either genotype. Altogether, these findings suggest that CFTR deficiency not only impair AP-1 signaling and pH-sensitive trafficking processes but also compromises vesicle trafficking and stress adaptation, ultimately altering host-virus interactions in the cystic fibrosis airway epithelium (**Table 5**).

**Table 5:** Differentially modulated processes identified by transcriptomic interaction analysis in SARS-CoV-2-infected CFBE41o^-^ WT and ΔF cells.

Functional category	WT CFBE41o^-^ cells	ΔF CFBE41o^-^ cells	Possible implication
Interferon response	Strong induction of type I/III IFNs (*IFNB1*, *IFNL1*–*3*), and ISGs (*IFITM1*–*3*, *OAS1*–*2*, *OASL*, *ISG15*)	Upregulated but less robust	Weakened antiviral signaling in CF
IL-6 signaling axis	Strong upregulation of IL-6 signaling axis (*ERPINB4*, *SMAD3*, *MUC5AC* and *ATP6V1C2*)	No significant induction	Lower inflammatory state in CF
Pathogen recognition/immune priming	Strong activation of *TLR4*, *DDX60*, *UBA7*	Lower induction	Less efficient viral sensing and immune activation
Endolysosomal acidification	High expression of *ATP6V1B1* (V-ATPase subunit)	Weak induction	Defective acidification in CF may impair viral entry and antigen presentation
Cytoskeletal remodeling	Strong upregulation of *MYOZ1*, *PRPH*	Reduced upregulation	Impaired intracellular trafficking and viral replication in CF
Early transcription factors	High expression of *FOS*, *FOSB*, *NR4A1*, *EGR2*	Expressed, but lower levels	Weaker and slower transcriptional activation in CF
Ion transport (K^+^ channels)	Robust upregulation of *KCNN1*	Modest induction	Impaired membrane polarization and signal transduction in CF
Metabolic and biosynthetic pathways	No major enrichment	Enriched (fat cell differentiation, vascular remodeling, ECM organization)	Shifted priorities in CF may reduce biosynthetic availability for viral replication
ER stress/UPR	Moderate activation of UPR genes (*HSPA5*, *ATF4*, *DDIT3*)	Attenuated activation	Less ER stress in CF may impair viral protein folding and assembly
Autophagy/senescence	Moderate activation	No significant induction	SARS-CoV-2 usurp wild-type autophagy and senescence pathway for its replication
Overall SARS-CoV-2 replication support	Multiple pathways supportive of viral replication are activated	Several critical replication-supporting pathways are blunted	CFTR dysfunction may limit SARS-CoV-2 replication and pathogenesis in CF cells

### 3.6 Corroboration of unique transcriptome regulation in response to SARS-CoV-2 in MucilAir™ WT and F508del^+/+^ models

To validate our findings in a model that better represents the human airway epithelium, we assessed transcriptomic response to SARS-CoV-2 infection in MucilAir™ F508del^+/+^ and WT patient-derived cell models (**Figure 1B**).

An initial comparison between CFBE41o^-^ and MucilAir™ cells was conducted to evaluate the consistency of their responses to SARS-CoV-2 infection. The analysis demonstrated that both cell models exhibit overlapping deregulated pathways and gene expression profiles following infection (**Supplementary Figure 1**), thereby supporting the use of MucilAir™ as a complementary system to confirm results obtained from CFBE41o^-^ cells.

**Figure 7:**
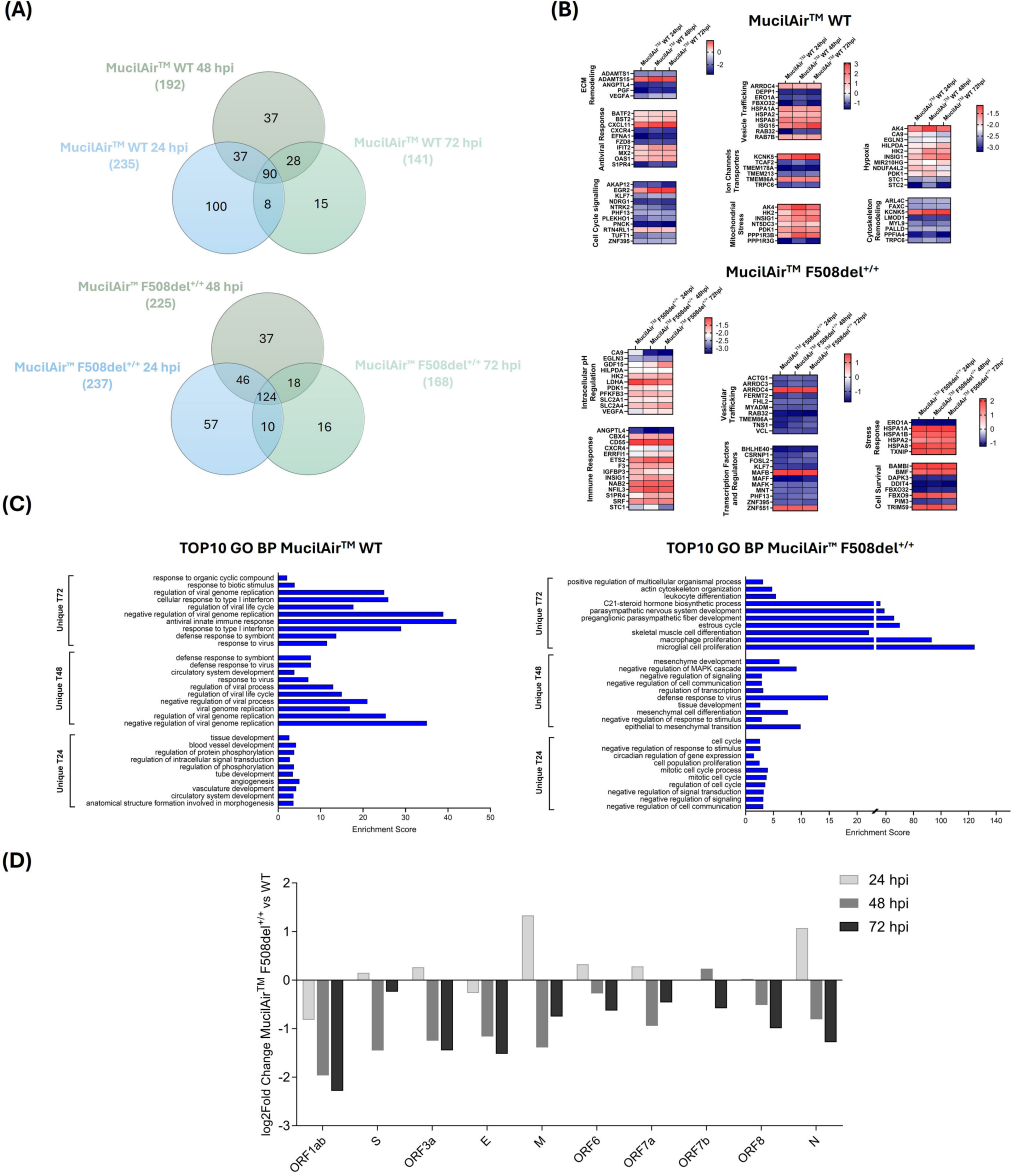
DEGs in response to SARS-CoV-2 infection in MucilAir™ WT and F508del^+/+^ at 24, 48 and 72 hpi. **(A)** Venn diagrams illustrate the overlap of DEGs between 24, 48 and 72 hpi in MucilAir™ WT (top) and in MucilAir™ F508del^+/+^ (bottom). **(B)** Heatmaps with expression profiles of DEGs common to both time points for MucilAir™ WT and F508del^+/+^, divided by their function. **(C)** Bar charts reporting the top 10 enriched GO terms for genes specifically regulated in MucilAir™ WT (left) and F508del^+/+^ (right). **(D)** Bar plots with the log_2_ fold change of viral gene expression at 24, 48, and 72 hpi in MucilAir™ F508del^+/+^ cells vs WT.

Following the same approach used for CFBE41o^-^ cells, we analyzed SARS-CoV-2-infected MucilAir™ cultures at 24, 48, and 72 hpi. Host responses included early metabolic and structural changes, followed by peak antiviral activity at 48 hpi, then a shift toward regulatory processes by 72 hpi (**Figure 7**).

As observed in cell lines, at 24 hpi viral gene expression was overall higher in F508del^+/+^ than in WT cells, suggesting enhanced viral entry in the CF background (**Figure 7D**). However, the expression levels became lower in F508del^+/+^ compared to WT cells at 48 hpi, a difference that persisted at 72 hpi, indicating in CF impaired viral replication or progression at later stages. Notably, *orf1ab* and *E* gene represented exceptions, as their expression levels were lower in F508del^+/+^ models compared with WT models at 24 hpi. This finding contrasts with the cell line model, where all viral genes exhibited increased expression in CF cells with respect to WT at early stages of infection. Such discrepancy may be explained by the cellular heterogeneity of primary airway cultures, where the contribution of different epithelial subtypes to infection dynamics could influence viral transcription profiles. In addition, the earlier restriction of *orf1ab* and *E* gene expression suggests that primary CF cells impose gene-specific limitations on viral replication and assembly.

Moreover, even in this cellular model, 48 hpi emerged as optimal to capture direct viral effects, driving our focus for subsequent analyses.

**Figure 8:**
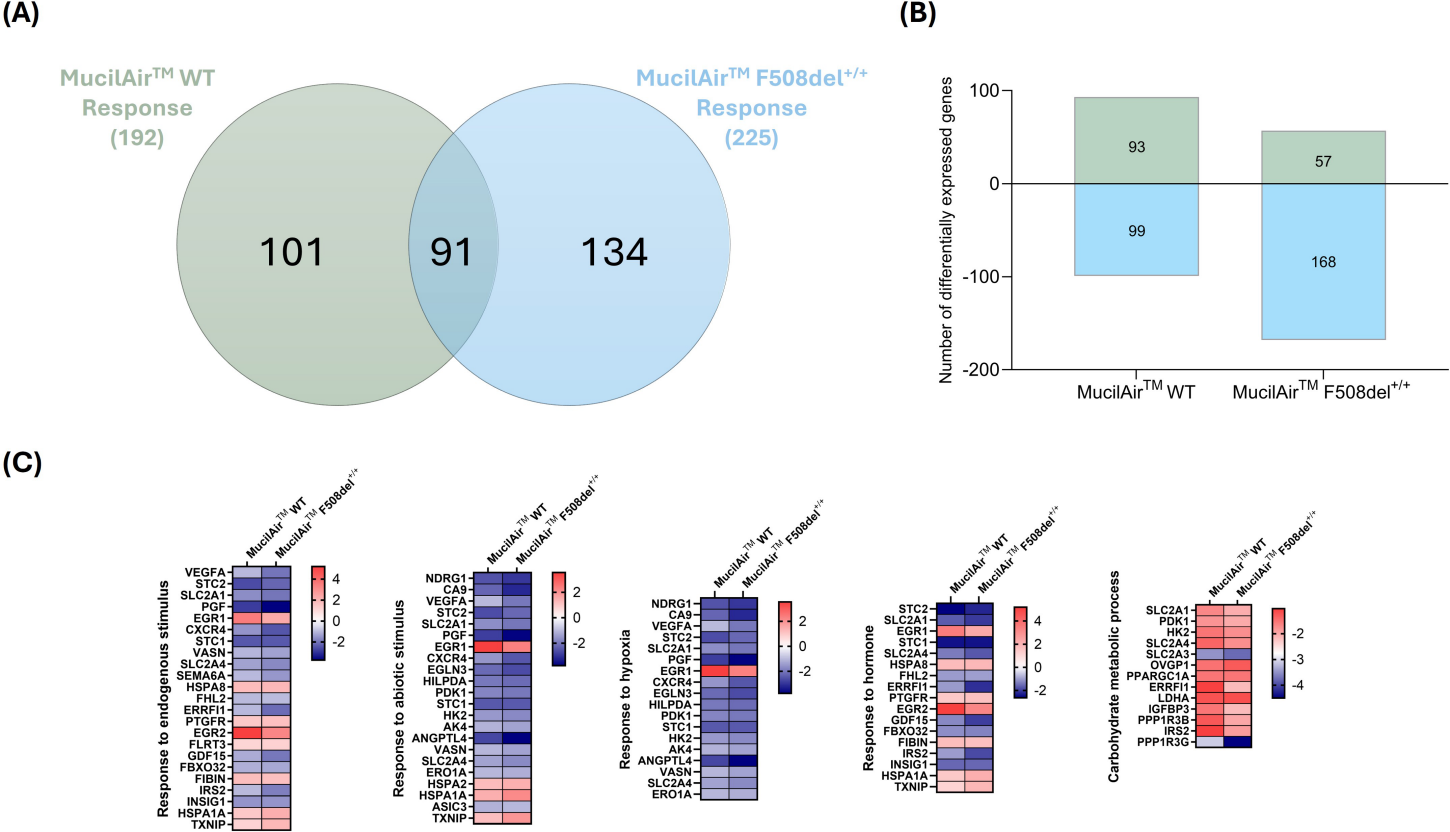
Comparative transcriptomic response to SARS-CoV-2 infection in MucilAir™ WT and F508del^+/+^. **(A)** Venn diagram comparing the differentially expressed (DEGs) genes between MucilAir™ WT (green; SARS-CoV-2 infected vs. mock-infected MucilAir™ WT) and MucilAir™ F508del^+/+^ (blue; SARS-CoV-2-infected vs. mock-infected MucilAir™ F508del^+/+^) response to SARS-CoV-2 infection. A total of 91 DEGs were common between WT and F508del^+/+^ response to viral infection. **(B)** The number (y-axis) and direction of change (upregulated = positive y-axis, downregulated = negative y-axis) of DEGs (Log_2_fold change>1.0, adjusted p-value < 0.05) of MucilAir™ WT and F508del^+/+^ response to SARS-CoV-2 infection (x-axis). **(C)** The relative expression of common DEGs divided by biological process (Log_2_fold change>1.0, adjusted p-value < 0.05) in SARS-CoV-2 infected WT and F508del^+/+^ MucilAir™.

Venn diagram at 48 hpi (**Figure 8A**) was used to compare genes that were uniquely and commonly modulated between F508del^+/+^ response and WT response to SARS-CoV-2 infection in MucilAir™. A total of 192 DEGs were observed in the MucilAir™ WT response to SARS-CoV-2, and 225 DEGs in the MucilAir™ F508del^+/+^ response (**Figure 8B**). Although there was considerable overlap between the groups (91 common DEGs), there were more unique DEGs specific to the MucilAir™ F508del^+/+^ response (134 DEGs) compared to the WT response (101 DEGs).

**Figure 9:**
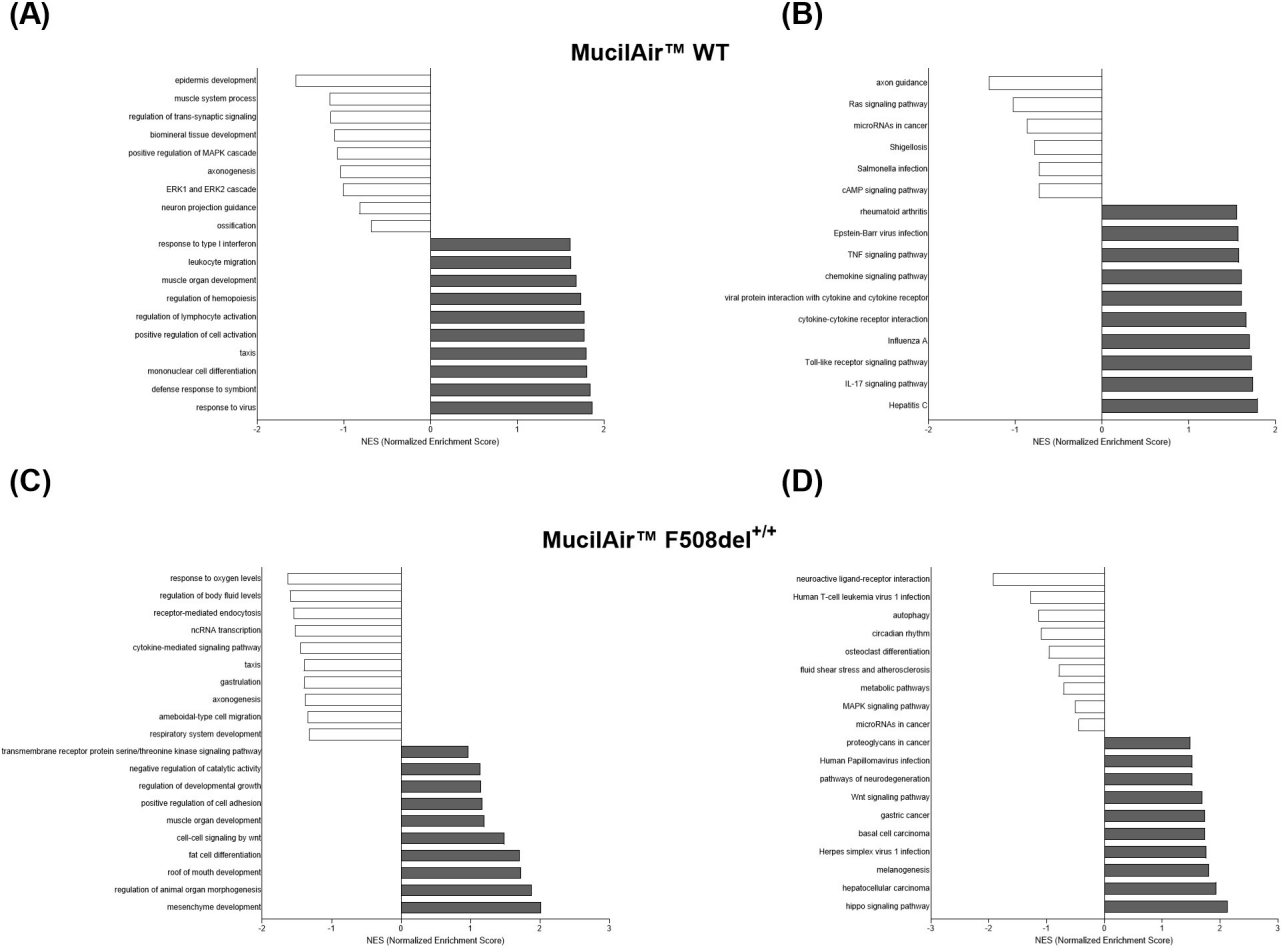
GSEA of unique DEGS in MucilAir™ WT and F508del^+/+^ response to SARS-CoV-2 infection. **(A)** Bar graphs of biological process GO analysis in MucilAir™ WT. **(B)** Bar graphs of KEGG pathways analysis in MucilAir™ WT. **(C)** Bar graphs of biological process GO analysis in MucilAir™ F508del^+/+^. **(D)** Bar graphs of KEGG pathways analysis in MucilAir™ F508del^+/+^.

The results confirmed a pivotal divergence in the WT and F508del^+/+^ MucilAir™ response to SARS-CoV-2, with differences emerging in immune regulation and pathways affected by intracellular pH. Although both cell types exhibited deregulation of metabolic pathways and responses to hypoxia, the magnitude and composition of the response were markedly attenuated in CF cells, especially for genes involved in immune response and cellular remodeling (**Figures 8C** and **9**).

Similar to the CFBE41o^-^ model, SARS-CoV-2 infection in MucilAir™ WT elicited a robust antiviral response, with marked upregulation of classical ISGs (*OAS1–3*, MX1/2, IFIT1–3, ISG15), chemokines (CXCL10, CXCL11, CCL5), and upstream sensors. This response was blunted in MucilAir™ F508del^+/+^, which showed limited ISG and chemokine induction.

In MucilAir™ cultures, SARS-CoV-2 infection induced deregulation of AP-1 transcription factors, which orchestrate oxidative stress, ER stress, and tissue remodeling responses. MucilAir™ WT showed robust upregulation of canonical AP-1 components (*FOS, FOSB, BATF2, EGR2, NR4A1*), reflecting activation of stress- and inflammation-responsive transcriptional programs. By contrast, CFTR-deficient models exhibited a globally repressed AP-1 profile, with downregulation of JUN, JUND, FOSL2, ATF3, and MAFF, indicative of impaired stress sensing and transcriptional activation.

Notably, CF models also upregulated developmental markers (WNT5A, SNAI2, LEF1), suggesting a shift toward tissue remodeling and a compensatory effort to preserve barrier integrity during infection.

ER stress and cytoskeletal responses were also differentially regulated. WT models activated canonical UPR genes (HSPA5, DDIT3), while F508del^+/+^ models showed a more limited or altered proteostasis response, marked by deregulation of HSPA1L, HSPA1B, ATF3, and DDIT4. Similarly, UPR activation was reduced in CFBE41o^-^ ΔF cells, potentially restricting ER-derived resources for viral replication. MucilAir™ WT cells also upregulated cytoskeletal and ion transport genes (*MYOZ1, PRPH*), supporting intracellular viral trafficking. These responses were absent in F508del^+/+^ model.

A striking difference was detected in the expression of genes involved in vesicular acidification and trafficking, processes critically dependent on intracellular pH and essential for viral entry and antigen presentation. WT models upregulated the V-ATPase subunit *ATP6V1B1*, while this response was absent in CF models. Genes for vesicle trafficking and endocytosis (ADM, TF, ARRDC3/4) were downregulated in F508del^+/+^ models indicating compromised endosomal sorting and intracellular routing of viral particles.

Unlike WT models, which upregulated the autophagy regulator SAMD9L, CF cells failed to induce this key gene. CF models also showed downregulation of stress and senescence and SASP-related markers like CDKN1C, ATF3, and DDIT4, indicating a blunted activation of these host defense mechanisms.

Analysis of cilia-related transcripts revealed selective differences between CFTR-deficient and WT cells. ELFN2 and CATSPER2 were downregulated, whereas the multiciliated cell fate regulator GMNC was upregulated in CF cells; junctional and polarity-associated genes (CLDN8, GJA4) were also differentially expressed. No induction of axonemal dyneins, intraflagellar transport components, or FOXJ1associated ciliogenesis markers were detected at baseline.

Following SARS-CoV-2 infection, cilia-related genes showed limited, gene-specific modulation in both WT and F508del^+/+^ MucilAir™ cultures. FLRT3 and EDAR were upregulated in both conditions, while CFTR-deficient cultures displayed reduced expression of LMOD1, IQCN, and DNHD1, indicating the absence of a broad ciliogenesis or axonemal remodeling program in either condition.

These findings highlight a fundamental difference in SARS-CoV-2 response between WT and CF epithelial cells (**Table 6**). WT models mount a strong antiviral response while activating processes that may support viral replication, including ER expansion, cytoskeletal remodeling, and autophagy. In contrast, CFTR-deficient models show a dampened immune and remodeling response, with increased stress adaptation and metabolic shifts, suggesting a less permissive environment for viral replication, potentially explaining the lower viral burden observed in some CF individuals.

**Table 6:** Differentially modulated biological processes in SARS-CoV-2-infected MucilAir™ WT and F508del^+/+^ cells.

Biological processes	MucilAir™ WT	MucilAir™ F508del^+/+^
Antiviral ISG response	Strong induction (*IFIT1–3*, *OAS1–3*, *MX1*, *ISG15*, *IFITM1*)	Largely absent or blunted
Proinflammatory cytokines/chemokines	High upregulation (e.g., *CXCL10*, *CXCL11*, *CCL5*, *TNFSF13B*)	Minimal or no induction
Innate immune pathways	Enrichment of *IFN-I/III*, *TNF*, *TLR*, *IL-17* signaling pathways	Poor enrichment of immune signaling
ER stress/unfolded protein response	Activation of canonical UPR genes (*HSPA5*, *ATF4*, *DDIT3*)	Altered ER stress profile (HSPA1B, ATF3, DDIT4), lacking full UPR activation
Vesicular acidification	Upregulated *ATP6V1B1* (V-ATPase subunit) supports viral entry and antigen processing	Little or no induction, consistent with known CFTR dysfunction
Cytoskeletal remodeling	Upregulated *MYOZ1*, *PRPH*, *KCNN1*: supports intracellular viral transport	No significant upregulation
Metabolic gene regulation	Mild suppression of metabolic genes	Strong downregulation, indicating energy conservation and stress adaptation
Tissue remodeling/development	Stable expression profile	↑ WNT5A, SNAI2, LEF1, BAMBI: mesenchymal remodeling, Wnt signaling activation
Oxygen sensing/hypoxia response	Moderately responsive (e.g., *PLAT*, *SRF*, *DDIT4*)	Broad downregulation, reduced hypoxia sensing
Autophagy	Increased expression of autophagy-related genes	No significant deregulation
Senescence	Upregulation of inflammatory SASP factors	Downregulation of key senescence regulators
Overall transcriptional strategy	Broad antiviral + proinflammatory response; supports both viral clearance and permissiveness	Blunted immune response; shift to homeostasis, metabolic buffering, limited viral support

### 3.7 qPCR genes validation shows high consistency with RNA-seq data

**Figure 10:**
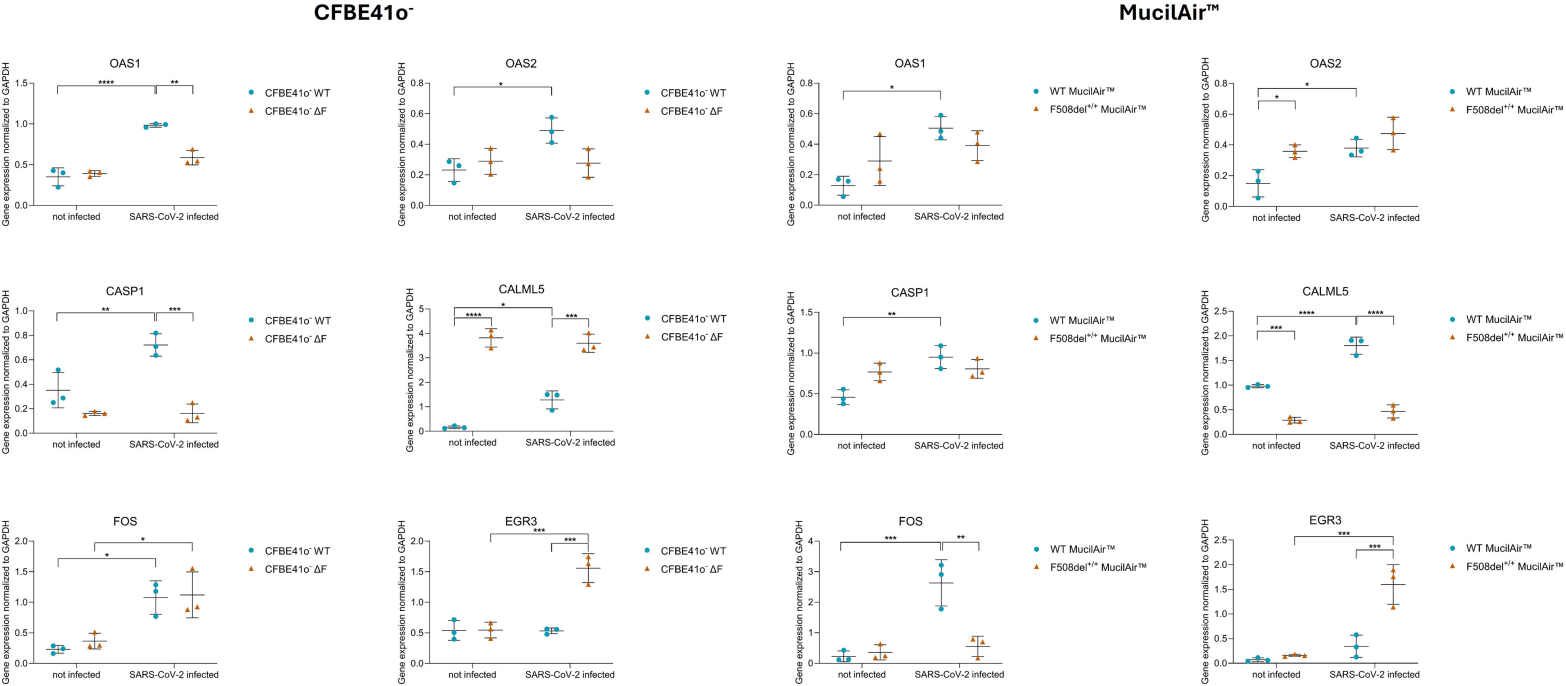
qPCR validation of selected top deregulated genes across independent pathways in CFBE41o^−^ (left) and MucilAir™ (right) models. Gene expression levels of*OAS1, OAS2, CASP1, CALML5, EGR3* and *FOS* were validated by qPCR in WT and CFTR-deficient cells following SARS-CoV-2 infection. Data points represent individual samples (n=3) for CFBE41o^−^ WT (circles) and ΔF (triangles), and for MucilAir™ WT (circles) and F508del^+^/^+^ (triangles).

Validation of RNA-seq data using RT-qPCR consistently confirmed the differential expression of key genes in both CFBE41o^-^ cells and the MucilAir™ models following SARS-CoV-2 infection (**Figure 10**). Specifically, *OAS1* and *OAS2*, which are crucial components of the interferon-induced antiviral response, along with *CASP1* and *CALML5*, involved in inflammation/pyroptosis and calcium signaling respectively, were significantly upregulated in infected WT CFBE41o^-^ cells, with only modest or no significant changes in ΔF cells. This distinct response highlights differences in antiviral and inflammatory pathway activation between WT and CFTR-deficient epithelial cells. In contrast, *FOS*, a component of the AP-1 transcription factor complex associated with stress and immune responses, exhibited significant upregulation in both WT and ΔF CFBE41o^-^ cells upon infection, indicating a shared activation of this pathway regardless of CFTR status. Interestingly, *EGR3*, a transcription factor implicated in immune regulation and cell proliferation, was uniquely and significantly upregulated in SARS-CoV-2-infected CFBE41o^-^ ΔF cells. These expression patterns were consistently observed in the MucilAir™ model as well, demonstrating strong agreement with both the initial RNA-seq data and the CFBE41o^-^ RT-qPCR validations, thus reinforcing the reliability and biological relevance of the observed transcriptional responses to SARS-CoV-2 infection across different cellular models. However, minor divergences in genes such as *CALML5* and *FOS* indicate model-specific transcriptional modulation, likely attributable to differences in epithelial composition and differentiation status between immortalized CFBE41o^−^ monolayers and primary, multicellular MucilAir™ epithelia.

## 4 Discussion

pwCF have experienced unexpectedly mild clinical outcomes following SARS-CoV-2 infection even though they were theoretically considered as a high-risk group. Supporting this observation, *in vitro* studies have consistently reported reduced viral replication in bronchial epithelial cells from pwCF (Bezzerri et al., 2023; Lagni et al., 2023; Lotti et al., 2022), raising the possibility that CFTR gene function/expression modulates the cellular environment affecting the viral ability to hijack host pathways. While the precise mechanisms remain incompletely understood, our findings shed light on several interlinked processes disrupted in CF cells that likely contribute to this protective phenotype.

Several mechanisms were suggested to explain this reduced clinical impact, especially in the context of the viral replication cycle, indicating that a reduction in specific SARS-CoV-2 receptors and changes in intracellular conditions such as pH may significantly affect the virion constitution and assembly.

Regarding SARS-CoV-2 susceptibility in CF, our RNA-seq data do not support major differences in *ACE2* or *TMPRSS2* mRNA expression between CFTR-WT and CFTR-ΔF models under basal conditions, nor following SARS-CoV-2 infection. These findings are consistent with previous reports indicating that reduced viral replication in CF is not directly linked to *ACE2* expression (Pagani et al., 2025) and with our observations showing comparable *ACE2* levels before infection, with only a late downregulation in WT cells emerging at 72 h post-infection (Lotti et al., 2022).

However, studies in primary CF airway epithelia have reported reduced *ACE2* expression at both mRNA and protein levels (Bezzerri et al., 2023), highlighting potential model-dependent differences in *ACE2* regulation that likely reflect epithelial differentiation status and cellular context. Taken together, the presence of divergent *ACE2* expression patterns across experimental systems suggests that *ACE2* regulation alone cannot be considered the main determinant of reduced SARS-CoV-2 replication in CF.

Altogether these data suggest that reduced viral replication in CF cells is not apparently due to differences in viral entry (even though additional studies are necessary to analyze the protein expression and their glycosylation) but rather arises from downstream limitations in viral exploitation of host machinery, largely driven by the altered intracellular environment characteristic of CF. This is also supported by viral genes expression analysis since viral gene expression in CF cells is highly present in the early time post-infection but features a sharp decline thereafter, while WT cells exhibited progressive increases. This reversal indicates that although entry may be favored in CF cells, their intracellular environment later limits replication, a result that is in line with our previous findings (Lagni et al., 2023).

A central role in CF different viral susceptibility compared to WT seems to be related to intracellular pH regulation. An effective SARS-CoV-2 replication relies on endosomal acidification to facilitate spike protein priming, membrane fusion, and the maturation of replication organelles such as double-membrane vesicles (DMVs) (Diehl and Schaal, 2013; Fares et al., 2025). WT cells infected with SARS-CoV-2 showed strong upregulation of genes regulating acidification in endosomes, lysosomes, and Golgi compartments (Bayati et al., 2021; Cortese et al., 2020). In stark contrast, CFTR-deficient cells exhibited lower basal expression and failed to induce pH-regulating genes like *SLC4A4* and *SLC7A14*, reflecting the chronic endolysosomal alkalinization known to accompany CFTR dysfunction. This pH dysregulation is likely to impair multiple stages of the viral life cycle, viral entry, uncoating, trafficking, and protein processing, while also compromising antigen presentation and immune activation (Teichgräber et al., 2008; Walsh and Naghavi, 2019).

Interestingly, disrupted acidification in CF cells also interferes with autophagy, a process SARS-CoV-2 subverts to generate replication organelles like DMVs (Gassen et al., 2021; Samimi et al., 2022). While WT cells activated numerous autophagy genes upon infection, ΔF cells showed minimal transcriptional response. This likely stems from pre-existing lysosomal dysfunction and impaired autophagic flux in CF, marked by Beclin-1 sequestration into aggresomes that block autophagosome formation (Lotti et al., 2023; Luciani et al., 2011, 2012). The loss of functional autophagy not only limits DMV generation but may also enhance viral degradation, limiting productive replication. Ultrastructural evidence supports these molecular findings: whereas WT cells formed mature DMVs and virus-containing vesicles, CFTR-deficient cells displayed irregular vesicles and features consistent with lysosomal degradation (Cortese et al., 2020; Merigo et al., 2022).

Finally, in accordance with a previous *in vitro* study (Merigo et al., 2024), WT cells but not ΔF cells, upregulated canonical markers of cellular senescence and the SASP upon infection. SARS-CoV-2 is indeed responsible for senescence induction and exacerbation of the SASP, and cellular senescence has been proposed as a critical regulator of SARS-CoV-2-evoked hyperinflammation (Gioia et al., 2023; Schmitt et al., 2022). The limited senescence response in CFTR-deficient cells, which already experience elevated stress signaling, may deprive SARS-CoV-2 of another pro-viral niche. Another major pathway exploited by SARS-CoV-2 is UPR, which facilitates ER remodeling and viral protein synthesis. WT cells upregulated canonical UPR markers (HSPA5, ATF4, DDIT3) upon infection. However, CF cells, already burdened by chronic ER stress due to misfolded CFTR, failed to mount this adaptive UPR (Trouvé et al., 2021). The ER dysfunction, together with impaired calcium signaling and disrupted ER–mitochondrial coupling (evidenced by *ASPH* downregulation) (Tong et al., 2017), limits the cellular capacity to support viral replication and assembly (Brewitz et al., 2020; Zou et al., 2018).

One of the most striking differences between genotypes was the immune response. As indicated in previous studies, the inflammatory genes are upregulated in CF mutated cells with respect to wild type cell lines (Mattoscio et al., 2025) at baseline thus explaining the hyperactivation of inflammation that is constantly detected in pwCF.

It is noteworthy that the SARS-CoV-2 infection elicited robust antiviral programs in WT cells marked by the induction of interferons, ISGs (Gao et al., 2023; Ortega-Prieto and Jimenez-Guardeño, 2024), and inflammatory mediators such as IL6, a key driver of cytokine release syndrome in severe COVID-19 (Ferreira-Gomes et al., 2021; Giamarellos-Bourboulis et al., 2020; Li et al., 2020; Rebendenne et al., 2021). In contrast, CFTR-deficient cells showed a significantly blunted immune response. This muted response aligns with several observations (Baresi et al., 2021; Oliva et al., 2025; Pagani et al., 2025) and may reflect chronic, baseline immune alteration in CF airways (Bitossi et al., 2021; Brazova et al., 2006; Kormann et al., 2017; O’Neal and Knowles, 2018), leading to a desensitized inflammatory state that could reduce the potential for cytokine-driven response upon infection (Prelli Bozzo et al., 2021).

Interestingly, CFTR-deficient cells demonstrate a dampened inflammatory profile, characterized by the suppression of IL6-associated signatures like SERPINB1, SMAD3, MUC5AC, LRG1, ATP6V1C2, and S100A9, consistent with findings by El-Husseini and colleagues (El-Husseini et al., 2023). This stands in stark contrast to the robust IL6-mediated cytokine activation observed in WT cells and other COVID-19 airway models (Ascierto et al., 2021; Brazova et al., 2006; Ferreira-Gomes et al., 2021; Giamarellos-Bourboulis et al., 2020; Gubernatorova et al., 2020; Li et al., 2020). Crucially, pwCF exhibit a naturally reduced level of IL6 in their respiratory tract, which may act as a protective factor against severe SARS-CoV-2 infection-related cytokine storms (Marcinkiewicz et al., 2020). This attenuated IL6 response in CF airway epithelium extends to other viral infections, with lower production reported even after rhinovirus and respiratory syncytial virus (RSV) exposure (Kieninger et al., 2012). Hence, SARS-CoV-2, while inducing high IL6 in healthy primary cells, induces only a minimal response in primary CF cells (Baresi et al., 2021; Bezzerri et al., 2023; Oliva et al., 2025). These cumulative observations strongly suggest that CFTR deficiency and its unique inflammatory state contribute to attenuated IL6 signaling in the airway.

Our RNA-seq analysis displayed several groups of differentially expressed genes, but intriguingly, the AP-1 complex is particularly influenced by CFTR and SARS-CoV-2 infection. AP-1 is a family of transcription factors formed by a homodimeric or heterocomplex of proteins belonging to Fos and Jun families. These factors are able to regulate several biological pathways including cell proliferation, inflammation, differentiation, and stress response. Our data demonstrated that at baseline, CFTR-deficient bronchial epithelial cells, both in CFBE41o^-^ and MucilAir™ cultures, did not exhibit significant differential expression of core AP-1 components compared with non-CF controls, indicating that the AP-1 transcriptional network remains largely quiescent under homeostatic conditions. Upon SARS-CoV-2 infection, WT cells mounted a robust AP-1 response, with strong induction of canonical immediate-early genes (*FOS, JUN, FOSB, ATF3, BATF2, EGR1-3, NR4A1*) that orchestrate inflammation, stress adaptation, and antiviral defense. In contrast, CF cells exhibited markedly attenuated AP-1 activation, consistent with a failure to engage virus-supportive transcriptional programs, which likely contributes to their reduced viral propagation. This blunted AP-1 response aligns with previous reports showing that SARS-CoV-2, as well as SARS-CoV, stimulates AP-1 via spike and ORF3a proteins to drive pro-inflammatory signaling, cytoskeletal remodeling, and cellular stress responses (He et al., 2003; Zhang et al., 2023), suggesting that CFTR deficiency imposes a cell-intrinsic limitation on these virus-induced pathways, shaping the distinctive antiviral and immunomodulatory phenotype of CF airway epithelium.

Given the central role of motile cilia in airway epithelial integrity, mucociliary clearance, and viral dissemination, we also examined whether SARS-CoV-2 infection induces modulation of cilia-related genes. In both CFBE41o^-^ cells and MucilAir™ cultures, cilia-associated transcripts showed only heterogeneous and gene-specific changes, including *FOXJ1, DNAH7, DNAH1, DAW1* and *TPPP3*. Our transcriptomic analyses, thus, did not reveal a coordinated deregulation of ciliogenesis, axonemal organization, or intraflagellar transport genes that would be indicative of impaired mucociliary clearance at 48 hpi. Instead, the data support a gene-specific modulation of cilia-associated transcripts, consistent with altered epithelial state rather than overt ciliary dysfunction. This pattern is consistent with reports showing that SARS-CoV-2 preferentially infects ciliated cells and induces ciliary damage, loss, or dedifferentiation rather than activation of ciliogenesis programs (Diambra et al., 2022; Robinot et al., 2021; Schreiner et al., 2022; Wang et al., 2022). Functional impairment of mucociliary clearance cannot be inferred from transcriptomic data alone and would require dedicated functional and ultrastructural analyses.

In summary, CFTR dysfunction is able to sustain an intracellular environment fundamentally hostile to SARS-CoV-2. Impaired acidification, attenuated IL6 signaling, chronic ER stress, disrupted organelle coordination, defective autophagy, and altered transcriptional responses can lead to inefficient viral entry, replication, and assembly. Among these, intracellular pH dysregulation emerges as a central mechanistic barrier, altering endosomal and lysosomal function, immune signaling, and vesicular remodeling, all essential for SARS-CoV-2 infection.

## 5 Conclusion

Our transcriptomic analysis demonstrates that CFTR dysfunction remodels the intracellular environment of airway epithelial cells in ways that are intrinsically hostile to SARS-CoV-2.

Rather than affecting viral receptor expression, CFTR deficiency disrupts intracellular processes, creating an environment that is intrinsically hostile to replication complex formation and virion assembly.

This study has, however, some limitations. Primary airway epithelial data were obtained from a limited number of MucilAir™ donors, and infections were performed exclusively with the ancestral Wuhan strain of SARS-CoV-2, which may restrict the generalizability of our findings to other viral variants or patient populations. In addition, our experimental models focused exclusively on the F508del CFTR mutation, the most prevalent mutation in cystic fibrosis. While this mutation represents the majority of CF cases, other CFTR variants may differentially affect intracellular homeostasis and virus-host interactions. Therefore, future studies that include a broader range of CFTR mutations will be important to assess the generalizability of these findings. Despite these constraints, this study not only enhances our understanding of virus-host interactions in CF but also uncovers host-intrinsic alterations providing a possible explanation for the attenuated viral phenotype observed in pwCF, and emphasizes the need to consider organellar homeostasis, particularly pH control, as a determinant of viral pathogenesis and potential therapeutic target.

## Data Availability

The datasets presented in this study can be found in online repositories. The names of the repository/repositories and accession number(s) can be found in the article/**Supplementary Material.**

## References

[B1] Abiatari I. Esposito I. De Oliveira T. Felix K. Xin H. Penzel R. . (2010). Moesin-dependent cytoskeleton remodelling is associated with an anaplastic phenotype of pancreatic cancer. J. Cell. Mol. Med. 14, 1166–1179. doi: 10.1111/J.1582-4934.2009.00772.X. PMID: 19432821 PMC3822753

[B2] Alessio N. Aprile D. Peluso G. Mazzone V. Patrone D. Di Bernardo G. . (2024). IGFBP5 is released by senescent cells and is internalized by healthy cells, promoting their senescence through interaction with retinoic receptors. Cell Commun. Signaling 22, 1–17. doi: 10.1186/S12964-024-01469-1. PMID: 38351010 PMC10863175

[B3] Ameen N. Silvis M. Bradbury N. A. (2006). Endocytic trafficking of CFTR in health and disease. J. Cystic Fibrosis: Off. J. Eur. Cystic Fibrosis Soc. 6, 1. doi: 10.1016/J.JCF.2006.09.002. PMID: 17098482 PMC1964799

[B4] Ascierto P. A. Fu B. Wei H. (2021). IL-6 modulation for COVID-19: The right patients at the right time? J. ImmunoTher. Cancer 9, 2285. doi: 10.1136/JITC-2020-002285. PMID: 33837054 PMC8042594

[B5] Badawi S. Ali B. R. (2021). *ACE2* nascence, trafficking, and SARS-CoV-2 pathogenesis: The saga continues. Hum. Genomics 15, 1–14. doi: 10.1186/S40246-021-00304-9. PMID: 33514423 PMC7844112

[B6] Barasch J. Kiss B. Prince A. Saiman L. Gruenert D. Ai-Awqati Q. (1991). Defective acidification of intracellular organelles in cystic fibrosis. Nature 352, 70–73. doi: 10.1038/352070A0. PMID: 1712081

[B7] Baresi G. Giacomelli M. Moratto D. Chiarini M. Conforti I. C. Padoan R. . (2021). Case report: Analysis of inflammatory cytokines IL-6, CCL2/MCP1, CCL5/RANTES, CXCL9/MIG, and CXCL10/IP10 in a cystic fibrosis patient cohort during the first wave of the COVID-19 pandemic. Front. Pediatr. 9. doi: 10.3389/FPED.2021.645063/BIBTEX PMC8291286 34295857

[B8] Bayati A. Kumar R. Francis V. McPherson P. S. (2021). SARS-CoV-2 infects cells after viral entry via clathrin-mediated endocytosis. J. Biol. Chem. 296. doi: 10.1016/j.jbc.2021.100306. PMID: 33476648 PMC7816624

[B9] Bebok Z. Collawn J. F. Wakefield J. Parker W. Li Y. Varga K. . (2005). Failure of cAMP agonists to activate rescued ΔF508 CFTR in CFBE41o– airway epithelial monolayers. J. Physiol. 569, 601. doi: 10.1113/JPHYSIOL.2005.096669. PMID: 16210354 PMC1464253

[B10] Bestle D. Heindl M. R. Limburg H. van Lam van T. Pilgram O. Moulton H. . (2020). *TMPRSS2* and furin are both essential for proteolytic activation of SARS-CoV-2 in human airway cells. Life Sci. Alliance 3. doi: 10.26508/LSA.202000786. PMID: 32703818 PMC7383062

[B11] Bezzerri V. Gentili V. Api M. Finotti A. Papi C. Tamanini A. . (2023). SARS-CoV-2 viral entry and replication is impaired in cystic fibrosis airways due to *ACE2* downregulation. Nat. Commun. 14, 1–15. doi: 10.1038/s41467-023-35862-0. PMID: 36627352 PMC9830623

[B12] Bitossi C. Frasca F. Viscido A. Oliveto G. Scordio M. Belloni L. . (2021). SARS-CoV-2 entry genes expression in relation with interferon response in cystic fibrosis patients. Microorganisms 9, 93. doi: 10.3390/MICROORGANISMS9010093. PMID: 33401565 PMC7824643

[B13] Blanco-Melo D. Nilsson-Payant B. E. Liu W. C. Uhl S. Hoagland D. Møller R. . (2020). Imbalanced host response to SARS-CoV-2 drives development of COVID-19. Cell 181, 1036–1045.e9. doi: 10.1016/J.CELL.2020.04.026. PMID: 32416070 PMC7227586

[B14] Bouhaddou M. Memon D. Meyer B. White K. M. Rezelj V. V. Correa Marrero M. . (2020). The global phosphorylation landscape of SARS-CoV-2 infection. Cell 182, 685–712.e19. doi: 10.1016/J.CELL.2020.06.034. PMID: 32645325 PMC7321036

[B15] Brazova J. Sismova K. Vavrova V. Bartosova J. Macek M. Lauschman H. . (2006). Polymorphisms of TGF-beta1 in cystic fibrosis patients. Clin. Immunol. 121, 350–357. doi: 10.1016/j.clim.2006.08.015. PMID: 17052957

[B16] Brewitz L. Tumber A. Schofield C. J. (2020). Kinetic parameters of human aspartate/asparagine–βhydroxylase suggest that it has a possible function in oxygen sensing. J. Biol. Chem. 295, 7826–7838. doi: 10.1074/JBC.RA119.012202. PMID: 32107312 PMC7278358

[B17] Bruscia E. Sangiuolo F. Sinibaldi P. Goncz K. K. Gruenert D. C. (2002). Isolation of CF cell lines corrected at ΔF508-CFTR locus by SFHR-mediated targeting. Gene Ther. 9, 683–685. doi: 10.1038/SJ.GT.3301741. PMID: 12032687

[B18] Caino M. C. Seo J. H. Wang Y. Rivadeneira D. B. Gabrilovich D. I. Kim E. T. . (2017). Syntaphilin controls a mitochondrial rheostat for proliferation-motility decisions in cancer. J. Clin. Invest. 127, 3755–3769. doi: 10.1172/JCI93172. PMID: 28891816 PMC5617650

[B19] Chen J. H. Cai Z. Sheppard D. N. (2009). Direct sensing of intracellular pH by the cystic fibrosis transmembrane conductance regulator (CFTR) Cl- channel. J. Biol. Chem. 284, 35495–35506. doi: 10.1074/jbc.M109.072678. PMID: 19837660 PMC2790979

[B20] Cheng C. M. Liu F. Li J. Y. Song Q. Y. (2018). DUSP1 promotes senescence of retinoblastoma cell line SO-Rb5 cells by activating AKT signaling pathway. Eur. Rev. Med. Pharmacol. Sci. 22, 7628–7632. doi: 10.26355/EURREV_201811_16377. PMID: 30536303

[B21] Collawn J. F. Matalon S. (2014). CFTR and lung homeostasis. Am. J. Physiol. Lung Cell. Mol. Physiol. 307, L917–L923. doi: 10.1152/AJPLUNG.00326.2014. PMID: 25381027 PMC4269691

[B22] Colombo C. Alicandro G. Dacco V. Gagliano V. Morlacchi L. C. Casciaro R. . (2021). SARS-CoV-2 infection in cystic fibrosis: A multicentre prospective study with a control group, Italy, February-July 2020. PloS One 16, e0251527. doi: 10.1371/JOURNAL.PONE.0251527. PMID: 33984027 PMC8118547

[B23] Cortese M. Lee J. Y. Cerikan B. Neufeldt C. J. Oorschot V. M. J. Köhrer S. . (2020). Integrative imaging reveals SARS-CoV-2-induced reshaping of subcellular morphologies. Cell. Host Microbe 28, 853–866.e5. doi: 10.1016/J.CHOM.2020.11.003. PMID: 33245857 PMC7670925

[B24] Cosgriff R. Ahern S. Bell S. C. Brownlee K. Burgel P. R. Byrnes C. . (2020). A multinational report to characterise SARS-CoV-2 infection in people with cystic fibrosis. J. Cystic Fibrosis 19, 355–358. doi: 10.1016/J.JCF.2020.04.012. PMID: 32376098 PMC7183287

[B25] Davis P. B. Drumm M. Konstan M. W. (1996). Cystic fibrosis. Am. J. Respir. Crit. Care Med. 154, 1229–1256. doi: 10.1164/AJRCCM.154.5.8912731. PMID: 8912731

[B26] Diambra L. Alonso A. M. Sookoian S. Pirola C. J. (2022). Single cell gene expression profiling of nasal ciliated cells reveals distinctive biological processes related to epigenetic mechanisms in patients with severe COVID-19. Comput. Biol. Med. 148, 105895. doi: 10.1016/J.COMPBIOMED.2022.105895. PMID: 35926268 PMC9338837

[B27] Diehl N. Schaal H. (2013). Make yourself at home: Viral hijacking of the PI3K/Akt signaling pathway. Viruses 5, 3192–3212. doi: 10.3390/V5123192. PMID: 24351799 PMC3967167

[B28] Ehrlich A. Uhl S. Ioannidis K. Hofree M. tenOever B. R. Nahmias Y. (2020). The SARS-CoV-2 transcriptional metabolic signature in lung epithelium. SSRN Electronic J. doi: 10.2139/SSRN.3650499. PMID: 40330906

[B29] El-Husseini Z. W. Khalenkow D. Lan A. van der Molen T. Brightling C. Papi A. . (2023). An epithelial gene signature of trans-IL-6 signaling defines a subgroup of type 2-low asthma. Respir. Res. 24. doi: 10.1186/S12931-023-02617-W. PMID: 38062491 PMC10704725

[B30] Fainardi V. Longo F. Chetta A. Esposito S. Pisi G. (2020). SARS-CoV-2 infection in patients with cystic fibrosian overwiew. Acta Biomedica 91, 1–5. doi: 10.23750/ABM.V91I3.10391. PMID: 32921729 PMC7716958

[B31] Fares P. Duhaini M. Tripathy S. K. Srour A. Kondapalli K. C. (2025). Acidic pH of early endosomes governs SARS-CoV-2 transport in host cells. J. Biol. Chem. 301. doi: 10.1016/j.jbc.2024.108144. PMID: 39732172 PMC11815683

[B32] Ferreira-Gomes M. Kruglov A. Durek P. Heinrich F. Tizian C. Heinz G. A. . (2021). SARS-CoV-2 in severe COVID-19 induces a TGF-β-dominated chronic immune response that does not target itself. Nat. Commun. 12, 1961–1961. doi: 10.1038/S41467-021-22210-3. PMID: 33785765 PMC8010106

[B33] Gao L. J. He Z. M. Li Y. Y. Yang R. R. Yan M. Shang X. . (2023). Role of OAS gene family in COVID-19 induced heart failure. J. Transl. Med. 21, 1–19. doi: 10.1186/S12967-023-04058-X/FIGURES/11 36949448 PMC10031198

[B34] Gassen N. C. Papies J. Bajaj T. Emanuel J. Dethloff F. Chua R. L. . (2021). SARS-CoV-2-mediated dysregulation of metabolism and autophagy uncovers host-targeting antivirals. Nat. Commun. 12, 1–15. doi: 10.1038/s41467-021-24007-w. PMID: 34155207 PMC8217552

[B35] Giamarellos-Bourboulis E. J. Netea M. G. Rovina N. Akinosoglou K. Antoniadou A. Antonakos N. . (2020). Complex immune dysregulation in COVID-19 patients with severe respiratory failure. Cell. Host Microbe 27, 992–1000.e3. doi: 10.1016/j.chom.2020.04.009. PMID: 32320677 PMC7172841

[B36] Gioia U. Tavella S. Martínez-Orellana P. Cicio G. Colliva A. Ceccon M. . (2023). SARS-CoV-2 infection induces DNA damage, through CHK1 degradation and impaired 53BP1 recruitment, and cellular senescence. Nat. Cell Biol. 25, 550–564. doi: 10.1038/S41556-023-01096-X. PMID: 36894671 PMC10104783

[B37] Gonçalves S. Yin K. Ito Y. Chan A. Olan I. Gough S. . (2021). COX2 regulates senescence secretome composition and senescence surveillance through PGE2. Cell Rep. 34, 108860. doi: 10.1016/J.CELREP.2021.108860. PMID: 33730589 PMC7972992

[B38] Gubernatorova E. O. Gorshkova E. A. Polinova A. I. Drutskaya M. S. (2020). IL-6: relevance for immunopathology of SARS-Cov-2. Cytokine Growth Factor Rev. 53, 13. doi: 10.1016/J.CYTOGFR.2020.05.009. PMID: 32475759 PMC7237916

[B39] Haggie P. M. Verkman A. S. (2009). Unimpaired lysosomal acidification in respiratory epithelial cells in cystic fibrosis. J. Biol. Chem. 284, 7681–7686. doi: 10.1074/JBC.M809161200. PMID: 19136560 PMC2658062

[B40] Hao S. Huang M. Xu X. Wang X. Song Y. Jiang W. . (2023). Identification and validation of a novel mitochondrion-related gene signature for diagnosis and immune infiltration in sepsis. Front. Immunol. 14. doi: 10.3389/FIMMU.2023.1196306. PMID: 37398680 PMC10310918

[B41] He R. Leeson A. Andonov A. Li Y. Bastien N. Cao J. . (2003). Activation of AP-1 signal transduction pathway by SARS coronavirus nucleocapsid protein. Biochem. Biophys. Res. Commun. 311, 870. doi: 10.1016/J.BBRC.2003.10.075. PMID: 14623261 PMC7111052

[B42] Ikeuchi M. Inoue M. Miyahara H. Sebastian W. A. Miyazaki S. Takeno T. . (2024). A pH imbalance is linked to autophagic dysregulation of inner ear hair cells in Atp6v1ba-deficient zebrafish. Biochem. Biophys. Res. Commun. 699, 149551. doi: 10.1016/J.BBRC.2024.149551. PMID: 38277730

[B43] Illek B. Maurisse R. Wahler L. Kunzelmann K. Fischer H. Gruenert D. C. (2008). Cl transport in complemented CF bronchial epithelial cells correlates with CFTR mRNA expression levels. Cell. Physiol. Biochem. 22, 57–68. doi: 10.1159/000149783. PMID: 18769032 PMC2927120

[B44] Itoh A. Uchiyama A. Taniguchi S. Sagara J. (2014). Phactr3/Scapinin, a member of protein phosphatase 1 and actin regulator (Phactr) family, interacts with the plasma membrane via basic and hydrophobic residues in the N-terminus. PloS One 9, e113289. doi: 10.1371/JOURNAL.PONE.0113289. PMID: 25405772 PMC4236165

[B45] Ji M. Li M. Sun L. Deng H. Zhao Y. G. (2023). DMV biogenesis during β-coronavirus infection requires autophagy proteins VMP1 and TMEM41B. Autophagy 19, 737–738. doi: 10.1080/15548627.2022.2103783. PMID: 35900889 PMC9851257

[B46] Jiang Y. Zhao T. Zhou X. Xiang Y. Gutierrez-Castrellon P. Ma X. (2022). Inflammatory pathways in COVID-19: Mechanism and therapeutic interventions. MedComm 3, e154. doi: 10.1002/MCO2.154. PMID: 35923762 PMC9340488

[B47] Kieninger E. Vareille M. Kopf B. S. Blank F. Alves M. P. Gisler F. M. . (2012). Lack of an exaggerated inflammatory response on virus infection in cystic fibrosis. Eur. Respir. J. 39, 297–304. doi: 10.1183/09031936.00054511. PMID: 21719483

[B48] Kormann M. S. D. Dewerth A. Eichner F. Baskaran P. Hector A. Regamey N. . (2017). Transcriptomic profile of cystic fibrosis patients identifies type I interferon response and ribosomal stalk proteins as potential modifiers of disease severity. PloS One 12. doi: 10.1371/JOURNAL.PONE.0183526. PMID: 28846703 PMC5573219

[B49] Kreidberg J. A. (2010). WT1 and kidney progenitor cells. Organogenesis 6, 61–70. doi: 10.4161/ORG.6.2.11928. PMID: 20885852 PMC2901809

[B50] Lagni A. Lotti V. Diani E. Rossini G. Concia E. Sorio C. . (2023). CFTR inhibitors display *in vitro* antiviral activity against SARS-CoV-2. Cells 12, 776. doi: 10.3390/CELLS12050776. PMID: 36899912 PMC10000629

[B51] Lee S. Yu Y. Trimpert J. Benthani F. Mairhofer M. Richter-Pechanska P. . (2021). Virus-induced senescence is a driver and therapeutic target in COVID-19. Nature 599, 283–289. doi: 10.1038/s41586-021-03995-1. PMID: 34517409

[B52] Li G. Fan Y. Lai Y. Han T. Li Z. Zhou P. . (2020). Coronavirus infections and immune responses. J. Med. Virol. 92, 424–432. doi: 10.1002/JMV.25685. PMID: 31981224 PMC7166547

[B53] Liang H. Luo D. Liao H. Li S. (2022). Coronavirus usurps the autophagy-lysosome pathway and induces membranes rearrangement for infection and pathogenesis. Front. Microbiol. 13. doi: 10.3389/FMICB.2022.846543. PMID: 35308399 PMC8924481

[B54] Liao Y. Wang J. Jaehnig E. J. Shi Z. Zhang B. (2019). WebGestalt 2019: gene set analysis toolkit with revamped UIs and APIs. Nucleic Acids Res. 47, W199–W205. doi: 10.1093/NAR/GKZ401. PMID: 31114916 PMC6602449

[B55] Lim Z. Q. Ng Q. Y. Oo Y. Chu J. J. H. Ng S. Y. Sze S. K. . (2021). Enterovirus‐A71 exploits peripherin and Rac1 to invade the central nervous system. EMBO Rep. 22. doi: 10.15252/EMBR.202051777. PMID: 33871166 PMC8183415

[B56] López-Domínguez J. A. Rodríguez-López S. Ahumada-Castro U. Desprez P. Y. Konovalenko M. Laberge R. M. . (2021). Cdkn1a transcript variant 2 is a marker of aging and cellular senescence. Aging 13, 13380–13392. doi: 10.18632/AGING.203110. PMID: 34035185 PMC8202863

[B57] Lotti V. Lagni A. Diani E. Sorio C. Gibellini D. (2023). Crosslink between SARS-CoV-2 replication and cystic fibrosis hallmarks. Front. Microbiol. 14. doi: 10.3389/FMICB.2023.1162470/XML PMC10213757 37250046

[B58] Lotti V. Merigo F. Lagni A. Di Clemente A. Ligozzi M. Bernardi P. . (2022). CFTR modulation reduces SARS-CoV-2 infection in human bronchial epithelial cells. Cells 11, 1347. doi: 10.3390/CELLS11081347. PMID: 35456026 PMC9028056

[B59] Lucas C. Wong P. Klein J. Castro T. B. R. Silva J. Sundaram M. . (2020). Longitudinal analyses reveal immunological misfiring in severe COVID-19. Nature 584, 463–469. doi: 10.1038/S41586-020-2588-Y. PMID: 32717743 PMC7477538

[B61] Luciani A. Villella V. R. Esposito S. Brunetti-Pierri N. Medina D. Settembre C. . (2010). Defective CFTR induces aggresome formation and lung inflammation in cystic fibrosis through ROS-mediated autophagy inhibition. Nat. Cell Biol. 12, 863–875. doi: 10.1038/NCB2090. PMID: 20711182

[B60] Luciani A. Villella V. R. Esposito S. Brunetti-Pierri N. Medina D. L. Settembre C. . (2011). Cystic fibrosis: a disorder with defective autophagy. Autophagy 7, 104–106. doi: 10.4161/AUTO.7.1.13987. PMID: 21048426

[B62] Luciani A. Villella V. R. Esposito S. Gavina M. Russo I. Silano M. . (2012). Targeting autophagy as a novel strategy for facilitating the therapeutic action of potentiators on ΔF508 cystic fibrosis transmembrane conductance regulator. Autophagy 8, 1657–1672. doi: 10.4161/AUTO.21483. PMID: 22874563 PMC3494594

[B63] Marat A. L. Haucke V. (2016). Phosphatidylinositol 3‐phosphates—at the interface between cell signalling and membrane traffic. EMBO J. 35, 561–579. doi: 10.15252/EMBJ.201593564. PMID: 26888746 PMC4801949

[B64] Marcinkiewicz J. Mazurek H. Majka G. Chain B. J. (2020). Are patients with lung cystic fibrosis at increased risk of severe and fatal COVID-19? Interleukin 6 as a predictor of COVID-19 outcomes. doi: 10.20452/pamw.15630, PMID: 33016684

[B65] Marin J. J. G. Serrano M. A. Herraez E. Lozano E. Ortiz-Rivero S. Perez-Silva L. . (2024). Impact of genetic variants in the solute carrier (SLC) genes encoding drug uptake transporters on the response to anticancer chemotherapy. Cancer Drug Resist. 7, 27. doi: 10.20517/CDR.2024.42. PMID: 39143954 PMC11322974

[B66] Martinez-Arroyo O. Selma-Soriano E. Ortega A. Cortes R. Redon J. (2021). Small Rab GTPases in intracellular vesicle trafficking: the case of Rab3A/Raphillin-3A complex in the kidney. Int. J. Mol. Sci. 22, 7679. doi: 10.3390/IJMS22147679. PMID: 34299299 PMC8303874

[B67] Mathew H. R. Choi M. Y. Parkins M. D. Fritzler M. J. (2021). Systematic review: cystic fibrosis in the SARS-CoV-2/COVID-19 pandemic. BMC Pulmon. Med. 21, 1–11. doi: 10.1186/S12890-021-01528-0/TABLES/4 PMC8135381 34016096

[B68] Mattoscio D. Baeza L. A. Bai H. Colangelo T. Castagnozzi S. Marzotto M. . (2025). Inflammation and epithelial-mesenchymal transition in a CFTR-depleted human bronchial epithelial cell line revealed by proteomics and human organ-on-a-chip. FEBS J. 292, 5086–5104. doi: 10.1111/FEBS.70050. PMID: 40029006 PMC12505456

[B69] McClenaghan E. Cosgriff R. Brownlee K. Ahern S. Burgel P. R. Byrnes C. A. . (2020). The global impact of SARS-CoV-2 in 181 people with cystic fibrosis. J. Cystic Fibrosis 19, 868–871. doi: 10.1016/J.JCF.2020.10.003. PMID: 33183965 PMC7641525

[B70] Merigo F. Lagni A. Boschi F. Bernardi P. Conti A. Plebani R. . (2024). Loss of CFTR reverses senescence hallmarks in SARS-CoV-2 infected bronchial epithelial cells. Int. J. Mol. Sci. 25, 6185. doi: 10.3390/IJMS25116185. PMID: 38892373 PMC11172982

[B71] Merigo F. Lotti V. Bernardi P. Conti A. Di Clemente A. Ligozzi M. . (2022). Ultrastructural characterization of human bronchial epithelial cells during SARS-CoV-2 infection: morphological comparison of wild-type and CFTR-modified cells. Int. J. Mol. Sci. 23, 9724. doi: 10.3390/IJMS23179724. PMID: 36077122 PMC9455986

[B72] Miao G. Zhao H. Li Y. Ji M. Chen Y. Shi Y. . (2021). ORF3a of the COVID-19 virus SARS-CoV-2 blocks HOPS complex-mediated assembly of the SNARE complex required for autolysosome formation. Dev. Cell 56, 427–442.e5. doi: 10.1016/j.devcel.2020.12.010. PMID: 33422265 PMC7832235

[B76] O’Neal W. K. Knowles M. R. (2018). Cystic fibrosis disease modifiers: complex genetics defines the phenotypic diversity in a monogenic disease. Annu. Rev. Genomics Hum. Genet. 19, 201–222. doi: 10.1146/ANNUREV-GENOM-083117-021329. PMID: 29709203

[B73] Ogando N. S. Dalebout T. J. Zevenhoven-Dobbe J. C. Limpens R. W. A. L. van der Meer Y. Caly L. . (2020). SARS-coronavirus-2 replication in Vero E6 cells: replication kinetics, rapid adaptation and cytopathology. J. Gen. Virol. 101, 925–940. doi: 10.1099/JGV.0.001453. PMID: 32568027 PMC7654748

[B74] Öhman T. Rintahaka J. Kalkkinen N. Matikainen S. Nyman T. A. (2009). Actin and RIG-I/MAVS signaling components translocate to mitochondria upon influenza A virus infection of human primary macrophages. J. Immunol. 182, 5682–5692. doi: 10.4049/JIMMUNOL.0803093. PMID: 19380815

[B75] Oliva J. Ruffin M. Calmel C. Gibeaud A. Pizzorno A. Gaudin C. . (2025). Divergent responses to SARS-CoV-2 infection in bronchial epithelium with pre-existing respiratory diseases. IScience 28, 111999. doi: 10.1016/J.ISCI.2025.111999. PMID: 40104058 PMC11914195

[B77] Ortega-Prieto A. M. Jimenez-Guardeño J. M. (2024). Interferon-stimulated genes and their antiviral activity against SARS-CoV-2. MBio 15, e02100–24. doi: 10.1128/MBIO.02100-24. PMID: 39171921 PMC11389394

[B78] Pagani I. Venturini A. Capurro V. Nonis A. Ghezzi S. Lena M. . (2025). Distinct responses of cystic fibrosis epithelial cells to SARS-CoV-2 and influenza A virus. Am. J. Respir. Cell Mol. Biol. 72, 308–319. doi: 10.1165/RCMB.2024-0213OC. PMID: 39311876 PMC11890075

[B79] Prelli Bozzo C. Nchioua R. Volcic M. Koepke L. Krüger J. Schütz D. . (2021). IFITM proteins promote SARS-CoV-2 infection and are targets for virus inhibition *in vitro*. Nat. Commun. 12, 1–13. doi: 10.1038/s41467-021-24817-y. PMID: 34321474 PMC8319209

[B80] Rao J. S. Bhoopathi P. Chetty C. Gujrati M. Lakka S. S. (2007). MMP-9 short interfering RNA induced senescence resulting in inhibition of medulloblastoma growth via P16INK4a and mitogen-activated protein kinase pathway. Cancer Res. 67, 4956–4964. doi: 10.1158/0008-5472.CAN-07-0380. PMID: 17510426 PMC1905835

[B81] Ratjen F. Bell S. C. Rowe S. M. Goss C. H. Quittner A. L. Bush A. (2015). Cystic fibrosis. Nat. Rev. Dis. Primers 1. doi: 10.1038/NRDP.2015.10. PMID: 27189798 PMC7041544

[B82] Ravindra N. G. Alfajaro M. M. Gasque V. Huston N. C. Wan H. Szigeti-Buck K. . (2021). Single-cell longitudinal analysis of SARS-CoV-2 infection in human airway epithelium identifies target cells, alterations in gene expression, and cell state changes. PloS Biol. 19, e3001143. doi: 10.1371/JOURNAL.PBIO.3001143. PMID: 33730024 PMC8007021

[B83] Rebendenne A. Chaves Valadão A. L. Tauziet M. Maarifi G. Bonaventure B. McKellar J. . (2021). SARS-CoV-2 triggers an MDA-5-dependent interferon response which is unable to control replication in lung epithelial cells. J. Virol. 95, e02415–20. doi: 10.1128/JVI.02415-20. PMID: 33514628 PMC8103705

[B84] Ribeiro C. M. P. Boucher R. C. (2010). Role of endoplasmic reticulum stress in cystic fibrosis-related airway inflammatory responses. Proc. Am. Thorac. Soc. 7, 387–394. doi: 10.1513/PATS.201001-017AW. PMID: 21030518 PMC3136959

[B85] Robinot R. Hubert M. Dias de Melo G. Lazarini F. Bruel T. Smith N. . (2021). SARS-CoV-2 infection induces the dedifferentiation of multiciliated cells and impairs mucociliary clearance. Nat. Commun. 12, 4354. doi: 10.1038/s41467-021-24521-x. PMID: 34272374 PMC8285531

[B86] Samimi N. Farjam M. Klionsky D. J. Rezaei N. (2022). The role of autophagy in the pathogenesis of SARS-CoV-2 infection in different cell types. Autophagy 18, 1728–1731. doi: 10.1080/15548627.2021.1989150. PMID: 34709967 PMC8567272

[B87] Scanlin T. F. Glick M. C. (2001). Glycosylation and the cystic fibrosis transmembrane conductance regulator. Respir. Res. 2, 276–279. doi: 10.1186/RR69/METRICS 11686896 PMC59516

[B88] Schmitt C. A. Tchkonia T. Niedernhofer L. J. Robbins P. D. Kirkland J. L. Lee S. (2022). COVID-19 and cellular senescence. Nat. Rev. Immunol. 23, 251–263. doi: 10.1038/s41577-022-00785-2. PMID: 36198912 PMC9533263

[B89] Schögler A. Stokes A. B. Casaulta C. Regamey N. Edwards M. R. Johnston S. L. . (2016). Interferon response of the cystic fibrosis bronchial epithelium to major and minor group rhinovirus infection. J. Cystic Fibrosis 15, 332–339. doi: 10.1016/j.jcf.2015.10.013. PMID: 26613982 PMC7185532

[B90] Schreiner T. Allnoch L. Beythien G. Marek K. Becker K. Schaudien D. . (2022). SARS-CoV-2 infection dysregulates cilia and basal cell homeostasis in the respiratory epithelium of hamsters. Int. J. Mol. Sci. 23, 5124. doi: 10.3390/IJMS23095124. PMID: 35563514 PMC9102945

[B91] Schulz B. L. Sloane A. J. Robinson L. J. Prasad S. S. Lindner R. A. Robinson M. . (2007). Glycosylation of sputum mucins is altered in cystic fibrosis patients. Glycobiology 17, 698–712. doi: 10.1093/GLYCOB/CWM036. PMID: 17392389

[B92] Stallaert W. Brüggemann Y. Sabet O. Baak L. Gattiglio M. Bastiaens P. I. H. (2018). Contact inhibitory Eph signaling suppresses EGF-promoted cell migration by decoupling EGFR activity from vesicular recycling. Sci. Signaling 11, 31. doi: 10.1126/SCISIGNAL.AAT0114/SUPPL_FILE/AAT0114_SM.PDF 30065026

[B93] Stanford G. E. Dave K. Simmonds N. J. (2021). Pulmonary exacerbations in adults with cystic fibrosis: a grown-up issue in a changing cystic fibrosis landscape. Chest 159, 93–102. doi: 10.1016/J.CHEST.2020.09.084. PMID: 32966813 PMC7502225

[B94] Teichgräber V. Ulrich M. Endlich N. Riethmüller J. Wilker B. De Oliveira-Munding C. C. . (2008). Ceramide accumulation mediates inflammation, cell death and infection susceptibility in cystic fibrosis. Nat. Med. 14, 382–391. doi: 10.1038/NM1748. PMID: 18376404

[B95] Tong M. Gao J.-S. Borgas D. de la Monte S. M. (2017). Phosphorylation modulates aspartyl-(asparaginyl)-β hydroxylase protein expression, catalytic activity and migration in human immature neuronal cerebellar cells. Cell. Biol.: Res. Ther. 6, 133. doi: 10.4172/2324-9293.1000133. PMID: 29607347 PMC5878085

[B96] Trouvé P. Férec C. Génin E. (2021). The interplay between the unfolded protein response, inflammation and infection in cystic fibrosis. Cells 10, 2980. doi: 10.3390/CELLS10112980. PMID: 34831204 PMC8616505

[B97] Venere M. Han Y. G. Bell R. Song J. S. Alvarez-Buylla A. Blelloch R. (2012). Sox1 marks an activated neural stem/progenitor cell in the hippocampus. Dev. (Cambridge) 139, 3938–3949. doi: 10.1242/DEV.081133. PMID: 22992951 PMC3472585

[B98] Vickers C. Hales P. Kaushik V. Dick L. Gavin J. Tang J. . (2002). Hydrolysis of biological peptides by human angiotensin-converting enzyme-related carboxypeptidase. J. Biol. Chem. 277, 14838–14843. doi: 10.1074/JBC.M200581200. PMID: 11815627

[B99] Walker N. M. Liu J. Stein S. R. Stefanski C. D. Strubberg A. M. Clarke L. L. (2016). Cellular chloride and bicarbonate retention alters intracellular pH regulation in Cftr KO crypt epithelium. Am. J. Physiol. Gastrointest. Liver Physiol. 310, G70–G80. doi: 10.1152/AJPGI.00236.2015. PMID: 26542396 PMC4719062

[B100] Walsh D. Naghavi M. H. (2019). Exploitation of cytoskeletal networks during early viral infection. Trends Microbiol. 27, 39–50. doi: 10.1016/j.tim.2018.06.008. PMID: 30033343 PMC6309480

[B101] Wang L. Liu C. Yang B. Zhang H. Jiao J. Zhang R. . (2022). SARS-CoV-2 ORF10 impairs cilia by enhancing CUL2ZYG11B activity. J. Cell Biol. 221, e202108015. doi: 10.1083/JCB.202108015. PMID: 35674692 PMC9184850

[B102] Wyler E. Mösbauer K. Franke V. Diag A. Gottula L. T. Arsiè R. . (2021). Transcriptomic profiling of SARS-CoV-2 infected human cell lines identifies HSP90 as target for COVID-19 therapy. IScience 24. doi: 10.1016/j.isci.2021.102151. PMID: 33585804 PMC7866843

[B103] Yoo J. S. Moyer B. D. Bannykh S. Yoo H. M. Riordan J. R. Balch W. E. (2002). Non-conventional trafficking of the cystic fibrosis transmembrane conductance regulator through the early secretory pathway. J. Biol. Chem. 277, 11401–11409. doi: 10.1074/jbc.M110263200. PMID: 11799116

[B105] Zhang L. Ramesh P. Atencia Taboada L. Roessler R. Zijlmans D. W. Vermeulen M. . (2024). UGT8 mediated sulfatide synthesis modulates BAX localization and dictates apoptosis sensitivity of colorectal cancer. Cell. Death Differ. 32, 657–671. doi: 10.1038/s41418-024-01418-y. PMID: 39580596 PMC11982410

[B104] Zhang J. Y. Whalley J. P. Knight J. C. Wicker L. S. Todd J. A. Ferreira R. C. (2023). SARS-CoV-2 infection induces a long-lived pro-inflammatory transcriptional profile. Genome Med. 15, 69. doi: 10.1186/S13073-023-01227-X. PMID: 37700317 PMC10498514

[B106] Zhen Y. Spangenberg H. Munson M. J. Brech A. Schink K. O. Tan K. W. . (2020). ESCRT-mediated phagophore sealing during mitophagy. Autophagy 16, 826–841. doi: 10.1080/15548627.2019.1639301. PMID: 31366282 PMC7158923

[B107] Zou Q. Hou Y. Wang H. Wang K. Xing X. Xia Y. . (2018). Hydroxylase activity of ASPH promotes hepatocellular carcinoma metastasis through epithelial-to-mesenchymal transition pathway. EBioMedicine 31, 287–298. doi: 10.1016/J.EBIOM.2018.05.004. PMID: 29764768 PMC6013968

