# Supplementary material for: Host transcriptomic analysis reveals a defective intracellular environment that limits SARS-CoV-2 replication in CFTR-deficient airway epithelium
Source: Front Cell Infect Microbiol. 2026 Mar 25;16:1754083. doi: 10.3389/fcimb.2026.1754083 (PMC13057564; doi:10.3389/fcimb.2026.1754083)
Supplement: Supplementary file 1 [file DataSheet1.docx]

Supplementary Material

## Supplementary Figures


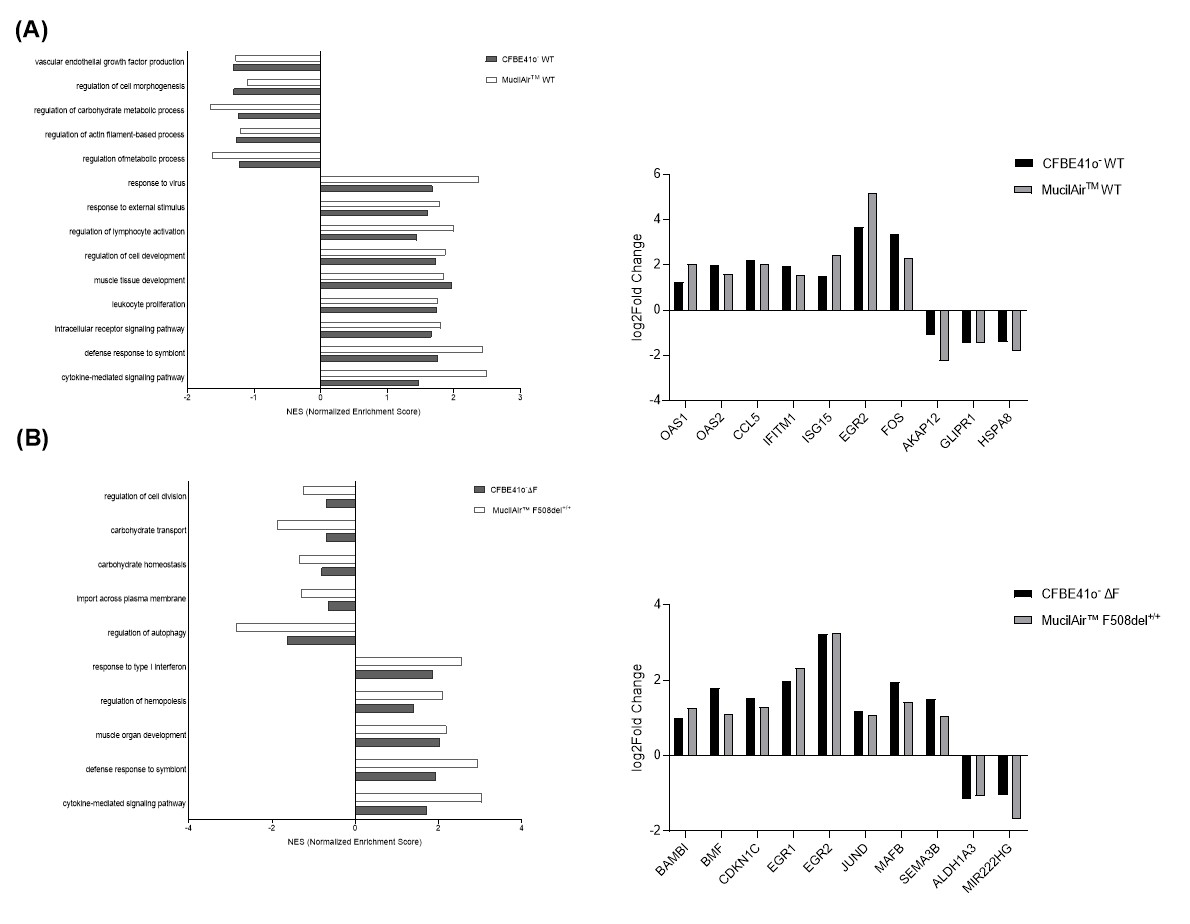


**Supplementary Figure 1.** Conserved transcriptional signatures between CFBE41o^-^ and MucilAir™ cell models. **(A)** Comparison of the most representative GO GSEA (on the left) and of the common TOP10 DEGs (on the right) between CFBE41o^-^ WT and MucilAir^TM^ WT. **(B)** Comparison of the most representative GO GSEA (on the left) and of the common TOP10 DEGs (on the right) between CFBE41o^-^ ΔF and MucilAir^TM^ F508del^+/+^
